# Benchmarking Orbital-Free Density-Potential Functional Theory of Electrified Metal-Solution Interfaces

**DOI:** 10.1021/acs.jctc.5c02180

**Published:** 2026-02-16

**Authors:** Chenkun Li, Xiwei Wang, Michael Eikerling, Jun Huang

**Affiliations:** † Institute of Energy and Climate Research, IET-3: Theory and Computation of Energy Materials, Forschungszentrum Jülich GmbH, 52425 Jülich, Germany; ‡ Faculty of Georesources and Materials Engineering, RWTH Aachen University, 52062 Aachen, Germany

## Abstract

The electrical double layer (EDL) at the metal–solution interface is a nanoscale region where quantum mechanical metal electrons meet almost classical electrolyte species. Describing metal electrons with orbital-free density-functional theory (DFT), the recently developed density-potential functional theoretical (DPFT) model constitutes a computationally efficient approach to modeling the EDL. However, the performance of orbital-free DFT is less studied for interfaces than for bulk phases. Herein, we develop a constant-potential Kohn–Sham–Poisson–Boltzmann theory with exact kinetic energy as a benchmark for DPFT models. Solving Kohn–Sham and Poisson–Boltzmann equations self-consistently, we obtain electron density, electrostatic potential, and double-layer capacitance of the EDL, which are then used to assess DPFT models with Thomas–Fermi–von Weizsäcker (TFvW) or Pauli-Gaussian kinetic energy functional. In general, TFvW outperforms the Pauli-Gaussian kinetic energy functional for modeling EDL. In addition, a much smaller gradient coefficient in the TFvW functional than the default value of 5/3 is suggested for modeling the EDL. These findings are instrumental to the future development of orbital-free DFT for electrochemical interfaces.

## Graphical abstract

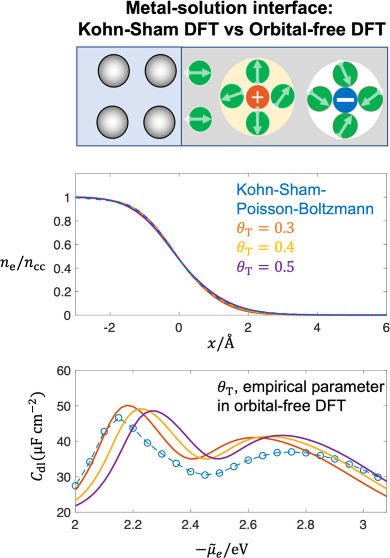

## Introduction

In response to the potential difference applied at the interface of a metal with an electrolyte solution, metal electrons, electrolyte ions and solvent molecules arrange into an electrical double layer (EDL). Understanding the microstructure and dynamics in the EDL is key to modulating the capacitive response and the charge transfer kinetics at the metal-solution interface. Modeling and computation are now an integral part of efforts that strive to unravel the properties of the nanoscale EDL, complementing experimental investigations.

Modeling of the EDL has a long history and comes in different flavors, see recent reviews.^1–3^ The classical Gouy–Chapman–Stern model conceptualizes the EDL as a serial connection of a rigid Helmholtz plane and a diffuse Gouy–Chapman layer.^4–6^ Historically, improvements to the Gouy–Chapman–Stern model have been made along three directions. First, the original point-charge description of electrolyte ions has been improved by considering steric ion effects,^7–9^ solvent polarization,^10,11^ and nonlocal short-range correlations.^12–14^ Second, the Helmholtz plane has been divided into an inner Helmholtz plane for specific adsorption and chemisorption^15,16^ and an outer Helmholtz plane for nonspecifically adsorbed ions. The first water layer at the IHP exerts a great influence on the double-layer capacitance.^17^ Third, the ideal-metal boundary in the original Gouy–Chapman–Stern model neglects the electron spillover and metal electronic effects. A jellium description of the metal has been devised to address this shortcoming.^15,16,18,19^ The metal is treated as an inhomogeneous electron gas, which is situated within a positive background charge contributed by the cationic cores of the metal. There exists a gap between the metal and the compartment of the electrolyte solution, which may shrink or expand as the electrode potential is changed.^16,20^

In the last three decades, the rapid growth in computing power has enabled fast-paced progress in density functional theory (DFT)-based models of the EDL.^21–25^ In a typical setup, these models represent the metal as a thin slab composed of a few layers of metal atoms, the electrolyte solution either as an implicit dielectric medium or explicitly as a thin water film with a few ions in *ab initio* molecular dynamics simulations or using a hybrid implicit/explicit scheme. These DFT-based models have greatly enhanced the atomistic understanding of the EDL, especially in the roles of interfacial water molecules in determining the potential of zero charge,^17,26,27^ the Helmholtz capacitance,^28–30^ and negative capacitance response induced by chemisorption of water.^31,32^ However, the high computation cost involved in solving the Kohn–Sham equations has limited DFT-based models to a periodic structure with a few hundreds of atoms. Therefore, the statistical sampling of the electrolyte solution is usually poor, resulting in an unsatisfactory description of the local reaction environment under constant potential conditions.

Despite the progress in classical Gouy–Chapman–Stern-type models and DFT-based models, modeling the EDL still faces two key challenges. The first one relates to treating electronic, ionic and solvent effects on an equal footing under constant potential conditions. The second one is about nonperiodic, mesoscopic EDLs met in many applications render first-principles calculation difficult. These challenges have driven us to develop a density-potential functional theoretical (DPFT) method.

The DPFT method combines orbital-free DFT for metal electrons and statistical field theory for the adjacent electrolyte solution. The grand potential of the EDL is then expressed as a hybrid functional of the electron density (*n*_e_) and the electrostatic potential (ϕ). Resulted from a coarse-grained description for the electrolyte solution, ϕ becomes a primary variable with an equal status as *n*_e_, contrasting the Kohn–Sham DFT where they are dual variables. This is why we refer to it as a DPFT approach. The fundamental idea behind the DPFT method is that potential should be treated as a primary variable in modeling electrochemical interfaces, contrasting current DFT models where potential effects are often added afterward as a correction term. The gap between the metallic and electrolytic compartments, which has been an enigma issue in previous jellium models,^18,19^ is naturally formed out of short-range interactions between metal electrons and solution particles described using empirical relations such as Morse potentials or Lennard-Jones potentials. The DPFT approach has contributed to understanding the two peaks of double-layer capacitance curves at silver electrodes,^33^ the solvent dependence of the potential of zero charge of Au (111)–organic solution interfaces,^34^ the short-range disjoining pressure between two metal surfaces,^35^ etc. The DPFT approach has demonstrated its effectiveness in describing the electro-ionic perturbations in the overlapping EDL at three-dimensional supported nanoparticles.^36^

A main shortcoming of the DPFT approach lies in the orbital-free approximation of the kinetic energy of metal electrons. Though orbital-free DFT has been carefully evaluated for atoms,^37^ molecules^38^ and bulk phases,^39^ its performance of EDLs that involve a large density gradient at the metal-solution interface is poorly known. Our goal here is to develop a first-principles based benchmarking approach for DPFT. To focus on this specific goal, we will consider an ideal metal/solution interface that is uniform in two dimensions, and exhibits heterogeneity only in the perpendicular direction.^40^ Therefore, the three-dimensional problem can be reduced to a one-dimensional problem. Metal electrons will be described using Kohn–Sham equations with exact kinetic energy for noninteracting electrons. Focusing on metal electronic effects, we will use a primitive model for the electrolyte solution, which is modeled as point charges dispersed in a dielectric continuum with a constant dielectric permittivity.^1^ Taken together, the above descriptions result in a Kohn–Sham–Poisson–Boltzmann theory of metal/solution interfaces. The Kohn–Sham–Poisson–Boltzmann equations are solved using an in-house self-consistent numerical scheme, serving as benchmark for calibrating orbital-free kinetic energy functionals in DPFT models.

The rest of the paper is organized as follows. We first review previous efforts on similar problems and identify the specific technical gaps that this work contributes to filling out. Second, we introduce the setup of Kohn–Sham–Poisson–Boltzmann and DPFT models, including the geometric structure, the governing equations, and the boundary conditions. Third, we propose a self-consistent numerical scheme that solves the coupled Kohn–Sham–Poisson–Boltzmann equations. Finally, we compare Kohn–Sham–Poisson–Boltzmann and DPFT models.

## Kohn–Sham–Poisson Models

Kohn–Sham–Poisson models have been developed for metal–vacuum systems^41–43^ and semiconductors^44,45^ to calculate the electronic structure. Regarded as an eigenvalue problem, the Kohn–Sham equation is solved using the eigenvalue method with the plane wave basis^46,47^ or atomic orbital basis,^48,49^ or the finite difference method.^50^ With the assumption of homogeneous electron gas in the bulk metal, the Kohn–Sham equation can be formulated as an ordinary differential equation. The Poisson equation is a second-order partial differential equation, which can be solved using numerical methods such as finite difference method for regular geometry,^51^ finite element method^52^ and finite volume method^53^ for irregular geometry. Solving coupled Kohn–Sham–Poisson equations is challenging. Charge distribution obtained from the Kohn–Sham equation may not conform to boundary conditions of the Poisson equation for the electrostatic potential. In the following, we review the progress in solving the Kohn–Sham-Poisson equations by formulating the Kohn–Sham equation as an ordinary differential equation and as an eigenvalue problem, respectively.

Lang and Kohn treated the Kohn–Sham equation as an ordinary differential equation to study charge densities, surface energies, and work functions of metal–vacuum surfaces.^42,54^ In their approach, the electron density was updated iteratively using a perturbational Newton–Raphson-type functional, which is more robust than simple fixed-point iteration. The electrostatic potential was calculated from direct integration using the Columbic potential expression, which is prone to numerical instability. Monnier and Perdew generalized the method of Lang and Kohn improving solution of electrostatic potential with an integral equation and further by considering high-order discrete-lattice perturbation potential δ*v* to study face-dependent surface energies, density profiles and work functions of nine simple metals.^55^ Their results can be reduced to those of Lang and Kohn in the limit of weak δ*v*. Subsequently, Yan^56^ and Della Sala^57^ employed the numerical routine of Lang and Kohn to solve the Kohn–Sham-Poisson equations and used it to calibrate the parameters of the orbital-free DFT. Posvyanskii and Shul’man improved the method of Lang and Kohn by introducing a predictor-corrector iteration scheme to update electron density.^58^ They further extended this scheme to a quasi-two-dimensional electron gas in the accumulation layer at the surface of a semiconductor with a degenerate electron gas in the bulk phase.^59^

The idea of treating the Kohn–Sham equation as an eigenvalue problem can be traced back to the Kohn–Sham DFT.^41^ To improve convergence of solving the Kohn–Sham–Poisson equations, many iteration schemes have been proposed. Stern systematically compared three different iteration methods of the electron density: including the fixed-convergence-factor method, the extrapolated-convergence-factor method, and the perturbational-iteration method to study semiconductor inversion layers.^60^ The last method is similar to the perturbational approach of Lang and Kohn.^42,54^ The fixed-convergence-factor method is now also called the linear mixing or under-relaxation method. Hamann introduced the Anderson acceleration^61^ into electronic structure calculation and studied semiconductor charge densities with hard-core and soft-core pseudopotentials.^62^ Around the same time, Pulay independently proposed an acceleration method of the direct inversion in the iterative subspace and used it for molecular calculations.^63^ Nowadays, the Anderson acceleration and Pulay’s method involve very similar algorithms. Dederichs and Zeller conducted a formal study of the linear mixing method in electronic structure calculations and compared its convergence with the Anderson acceleration for 3*d* impurities in Cu.^64^ Using the predictor-corrector iteration method, Luscombe et al. studied the electron confinement in the GaAs quantum wire by solving the coupled Kohn–Sham–Poisson equations, and compared the results with those obtained from the Thomas–Fermi theory.^45^ After that, Trellakis et al. systematically compared the convergence of the linear mixing method and the predictor–corrector method of solving the two-dimensional Kohn–Sham–Poisson equations in a GaAs-AlGaAs-based quantum wire.^65^ They found that the predictor-corrector method converged much faster than the linear mixing method and the exchange-correlation potential impaired both convergence and stability. Recently, Gil-Corrales et al. studied the electronic properties of GaAs quantum well wires with various cross-sectional shapes using the predictor-corrector iteration method.^66^ A more comprehensive summary and discussion on iteration methods of solving the coupled Kohn–Sham–Poisson equations can be found in recent review article by Novák et al.^67^

Although many studies about self-consistent solutions of the Kohn–Sham–Poisson equations exist, they have been focusing on the metal-vacuum interface or the semiconductor system under electroneutrality. The self-consistent solution of Kohn–Sham–Poisson–Boltzmann equations for metal-solution interfaces under constant potential conditions has not been reported to the best of our knowledge. In this work, we propose a method to solve Kohn–Sham–Poisson–Boltzmann equations under constant potential conditions, and use it to assess the accuracy of DPFT where the kinetic energy of electron densities is described using the Thomas–Fermi–von Weizsäcker^68–70^ and Pauli–Gaussian kinetic energy functionals.^39^

## Model Setup

In this section, we specify the computational domain tailored to focus on metal electronic effects. Thereafter, we introduce the governing equations and their boundary conditions for both Kohn–Sham–Poisson–Boltzmann and DPFT models. Subsequently, we make a nondimensionalization for governing equations. In the following, we specify how to achieve constant-potential conditions in Kohn–Sham–Poisson–Boltzmann and DPFT models, and develop a numerical flowchart for self-consistently solving the Kohn–Sham–Poisson–Boltzmann model. Finally, we parametrize the models and calculate and analyze the solutions obtained.

### 1D Symmetrical Geometry

We consider a 1D symmetrical geometry consisting of a metallic slab in the middle of two electrolyte solutions, as shown in Figure 1. The symmetrical geometry simplifies the boundary conditions for electronic wave functions. The treatment of boundary conditions of wave functions for an asymmetrical geometry was discussed in earlier studies.^42,54^ Wave functions are assumed to be uniform in the other two dimensions, where translational symmetry is preserved. This symmetry allows the wave functions to be represented as plane waves parallel to the surface, while the variation along the *x* direction (normal to the interface) is treated explicitly. Therefore, the original 3D problem is reduced to a 1D problem exhibiting variations only in the coordinate normal to the metal surface (the detailed formulation is provided in supplementary note 1 in the Supporting Information).

**Figure 1:**
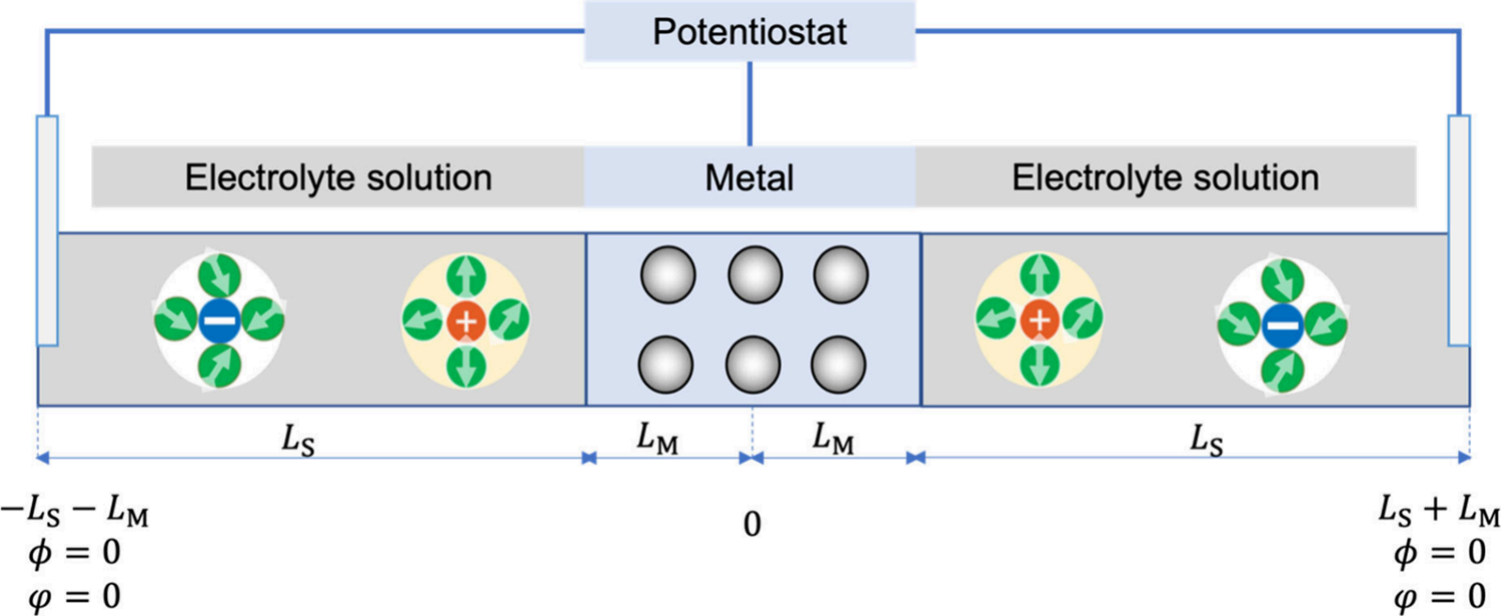
Schematic diagram of 1D planar symmetrical geometry consisting of a metallic slab in the middle of two electrolyte solutions. *L*_M_ and *L*_S_ are the dimensionless thicknesses of the metal and electrolyte solution, respectively.

### Governing Equations

#### Kohn–Sham–Poisson–Boltzmann Models

The governing equations for the Kohn–Sham–Poisson–Boltzmann model consist of the Kohn–Sham equation and Poisson–Boltzmann equation.

The 1D Kohn–Sham equation for a system of fictitious noninteracting electrons, subjected to an effective potential *v*_eff_ can be expressed as^41^

1 $$\left[-\frac{\hbar^{2}}{2m_{e}}\frac{d^{2}}{dx^{2}}+v_{\mathrm{eff}}\left(x\right)\right]\varphi_{i}\left(x\right)=\varepsilon_{i}\varphi_{i}\left(x\right)$$

where ℏ is the reduced Planck constant, *m*_e_ is the mass of an electron, ε_*i*_ is the energy, φ_*i*_ is the wave function, and *v*_eff_ is the effective external potential energy given by

2 $$v_{\mathrm{eff}}\left(x\right)=-e_{0}\phi \left(x\right)+v_{\mathrm{XC}}\left(x\right)$$

where the first term is the electrostatic potential energy with ϕ­(*x*) being the local electrostatic potential and *e*_0_ the elementary charge of an electron. Herein, −*e*_0_ϕ­(*x*) already includes the contributions of the Hartree potential energy and external potential energy, namely, −*e*_0_ϕ­(*x*) = *v*_H_ + *v*_ex_. *v*_XC_(*x*) is the exchange-correlation (XC) potential energy, which is a function of local electron density *n*_e_(*x*) and its gradient ∇*n*_e_(*x*). For the base case, we use the primitive *v*_XC_(*x*), namely, the Dirac–Wigner functional^70^ with the local density approximation:

3 $$v_{\mathrm{XC}}\left(x\right)=e_{\mathrm{au}}a_{0}\left(\frac{4}{3}c_{3}n_{e}^{1/3}\left(x\right)+c_{4}n_{e}^{1/3}\left(x\right)\frac{\left(\frac{4}{3}c_{5}+a_{0}n_{e}^{1/3}\left(x\right)\right)}{{\left(c_{5}+a_{0}n_{e}^{1/3}\left(x\right)\right)}^{2}}\right)$$

where $e_{\mathrm{au}}=\frac{e_{0}^{2}}{4\pi \epsilon_{0}a_{0}}$ is the atomic energy with Bohr radius *a*_0_ and vacuum permittivity ϵ_0_. The first term in the bracket, with $c_{3}=-\frac{3}{4}{\left(\frac{3}{\pi }\right)}^{1/3}$ and electron density *n*_e_, represents the exchange potential of a uniform electron gas; the second term, with *c*_4_ = – 0.056, *c*_5_ = 0.079, is Wigner’s expression for the correlation potential.^71^

The 1D Poisson–Boltzmann equation obtained from the DPFT functional can be expressed as (the detailed derivation is provided in supplementary note 2 in the Supporting Information):^72^

4 $$-\nabla \left(\epsilon_{\mathrm{op}}\nabla \phi \left(x\right)\right)=e_{0}\left(n_{\mathrm{cc}}-n_{e}\right)+e_{0}\left(q_{c}n_{c}-q_{a}n_{a}\right)$$

where ϵ_op_ is the dielectric permittivity, *n*_cc_ is the metal catonic core density, *n*_c_/*n*_a_ is the cation/anion density in the solution, and *q*_c_/*q*_a_ is the absolute value of cation/anion valence.

*n*_c_/*n*_a_ is described using the modified Boltzmann equation that incorporates ion size effect via a lattice-gas description:^1,7^

5 $$n_{c,a}=n_{c,a}^{\mathrm{bulk}}\frac{\exp\left(-\frac{\left(w_{c,a}\pm e_{0}q_{c,a}\phi \right)}{k_{B}T}\right)}{\chi_{c}\exp\left(-\frac{\left(e_{0}q_{c}\phi +w_{c}\right)}{k_{B}T}\right)+\chi_{a}\exp\left(\frac{e_{0}q_{a}\phi -w_{a}}{k_{B}T}\right)+1-\chi_{c}-\chi_{a}}$$

where *n*_c,a_^bulk^ is the cation/anion density in the solution bulk, *k*_B_ is the Boltzmann constant, *T* is the temperature, χ_c,a_ = *n*_c,a_^bulk^ *a* ^3^ is the bulk volume fraction of ions, with *a* being the ion size, and *w*_c,a_ is the Morse potential that describes short-range interactions between metal and electrolyte solution. We use the repulsive part of *w*_c,a_ to prevent solution cations/anions from penetrating into the metal, expressed as:^73^

6 $$w_{c,a}=D_{l}\exp\left(-2\beta_{c,a}\left(d-d_{c,a}\right)\right)$$

where *D*_l_ is the strength of the Morse potential, β_c,a_ is the coefficient controlling the decay of the Morse potential, *d* and *d*_c,a_ denote the distances from the position and equilibrium position of cation/anion to the metal surface, respectively.

The Kohn–Sham and Poisson–Boltzmann equations are coupled via ϕ and *n*_e_. The Kohn–Sham equation solves for φ_*i*_ and ε_*i*_ rather than *n*_e_, therefore, we need to calculate *n*_e_ from φ_*i*_ and ε_*i*_,^74,75^

7 $$n_{e}=\frac{1}{\pi }\frac{m_{e}k_{B}T}{\hbar^{2}}\Sigma {\left|\varphi_{i}\left(x\right)\right|}^{2}\ln\left(1+e^{{\mathrm{\mu ̃}}_{e}-\varepsilon_{i}/k_{B}T}\right)$$

where μ̃_e_ is the electrochemical potential of electrons, equivalent to the Fermi level. μ̃_e_ is usually defined as μ̃_e_ = μ_e_ – *e*_0_ϕ, where μ_e_ is the chemical potential composed of the kinetic energy and exchange-correlation potential energy, namely, $\mu_{e}=e_{\mathrm{au}}\left(\frac{\delta t_{\mathrm{kin}}}{\delta n_{e}}+\frac{\delta u_{\mathrm{XC}}}{\delta n_{e}}\right)$ . The detailed derivation of *n*_e_ is provided in supplementary note 5 in the Supporting Information.

#### DPFT Models

The governing equations for the DPFT model consist of an equation describing the electron density and the Poisson–Boltzmann equation. The latter is the same as that in the Kohn–Sham–Poisson–Boltzmann model.

For the DPFT model using the Thomas–Fermi–von Weizsäcker (TFvW) kinetic energy functional, a variational analysis of the grand potential *g* with respect to *n*_e_ using the Euler–Lagrange equation gives:

8 $$\frac{\partial g}{\partial n_{e}}-\nabla \left(\frac{\partial g}{\partial \nabla n_{e}}\right)=0$$

where *g* is the volumetric grand potential of the electric double layer shown in eq (S16) in the Supporting Information.

After algebraic manipulations provided in supplementary note 3 in the Supporting Information, we obtain the controlling equation of the electron density:^33^

9 $$\mathrm{\nabla ̅}\mathrm{\nabla ̅}{\mathrm{n̅}}_{e}=\frac{20}{3}{\mathrm{n̅}}_{e}\frac{1}{\theta_{T}}\left(\frac{\partial t_{\mathrm{TF}}}{\partial {\mathrm{n̅}}_{e}}+\frac{\partial v_{\mathrm{XC}}}{\partial {\mathrm{n̅}}_{e}}-\frac{e_{0}\phi +{\mathrm{\mu ̃}}_{e}}{e_{au}}\right)+\frac{2}{3}\frac{1}{{\mathrm{n̅}}_{e}}{\left(\mathrm{\nabla ̅}{\mathrm{n̅}}_{e}\right)}^{2}$$

where θ_T_ is the coefficient of gradient terms in kinetic energy, $t_{\mathrm{TF}}=\frac{3}{10}{\left(3\pi^{2}\right)}^{2/3}{\left(n_{e}a_{0}^{3}\right)}^{5/3}$ is the Thomas–Fermi kinetic energy functional, *μ̃*_e_ is the electrochemical potential of electrons, ∇̅ = *a*_0_∇ is the dimensionless Laplace operator, and *n̅*_e_ = *n*_e_ *a*_0_^3^ is the dimensionless electron density.

For the DPFT model using the Pauli–Gaussian kinetic energy functional, we obtain a similar equation for the electron density:

10 $$\mathrm{\nabla ̅}\mathrm{\nabla ̅}{\mathrm{n̅}}_{e}=\frac{20}{3}{\mathrm{n̅}}_{e}\frac{1}{\left(\theta_{T}-{\mu_{\mathrm{PG}}e}^{-\mu_{\mathrm{PG}}s^{2}}\right)}\left(e^{-\mu_{\mathrm{PG}}s^{2}}\frac{\partial t_{\mathrm{TF}}}{\partial {\mathrm{n̅}}_{e}}+\frac{\partial v_{\mathrm{XC}}}{\partial {\mathrm{n̅}}_{e}}-\frac{\left(e_{0}\phi +{\mathrm{\mu ̃}}_{e}\right)}{e_{au}}\right)+\frac{\left(\theta_{T}+\frac{2}{3}{\mu e}^{-\mu_{\mathrm{PG}}s^{2}}+\frac{16}{3}{{{\mu_{\mathrm{PG}}}^{2}s}^{2}e}^{-\mu_{\mathrm{PG}}s^{2}}\right)}{2{\mathrm{n̅}}_{e}\left(\theta_{T}-{\mu_{\mathrm{PG}}e}^{-\mu_{\mathrm{PG}}s^{2}}\right)}{\left(\mathrm{\nabla ̅}{\mathrm{n̅}}_{e}\right)}^{2}$$

where $s=\frac{\left|\nabla n_{e}\right|}{{2\left({3\pi }^{2}\right)}^{1/3}n_{e}^{4/3}}$ is the reduced gradient term, and μ_PG_ is a coefficient in the Pauli–Gaussian functional. A detailed derivation of eq 10 is provided in the supplementary note 4 in the Supporting Information. The Pauli–Gaussian functional is reduced to the TFvW kinetic energy functional when μ_PG_ = 0, and eq 10 is reduced to eq 9.

### Boundary Conditions

Boundary conditions to close the Kohn–Sham and Poisson–Boltzmann equations are shown in Figure 1,

11 φ=0

12 ϕ=0

which means that the wave function cannot propagate into the bulk solution and the electrostatic potential in the bulk solution is taken as a reference, see more discussion in supplementary note 6 in the Supporting Information.

For the governing equation of the electron density in the DPFT, the boundary condition is as follows,

13 $$n_{e}=0$$

which means that metal electrons are absent in the bulk solution.

### Nondimensionalization

To facilitate numerical solutions, we define dimensionless variables, marked with an overbar, as follows:

14 $$\begin{array}{c} \mathrm{x̅}=\frac{x}{a_{0}},\mathrm{\nabla ̅}=a_{0}\nabla ,{\mathrm{n̅}}_{l}=\frac{n_{l}}{a_{0}^{-3}},{\mathrm{\epsilon ̅}}_{\mathrm{op}}=\frac{\epsilon_{\mathrm{op}}}{\epsilon_{0}}\\ {\mathrm{v̅}}_{\mathrm{eff}}=\frac{v_{\mathrm{eff}}}{k_{B}T}=-\mathrm{\phi ̅}+{\mathrm{v̅}}_{\mathrm{XC}},\mathrm{\varepsilon ̅}=\frac{\varepsilon }{k_{B}T},{\overset{¯}{\overset{\sim }{\mu }}}_{e}=\frac{{\mathrm{\mu ̃}}_{e}}{k_{B}T},\mathrm{\phi ̅}=\frac{\phi }{k_{B}T/e_{0}} \end{array}$$

We obtain the following dimensionless Kohn–Sham and Poisson–Boltzmann equations:

15 [−12ℏ2ma02kBTd2dx̅2+v̅eff]φ=εφ

16 $$-\mathrm{\nabla ̅}\left({\mathrm{\epsilon ̅}}_{\mathrm{op}}\mathrm{\nabla ̅}\mathrm{\phi ̅}\right)=\frac{4\pi e_{au}}{k_{B}T}\left({\mathrm{n̅}}_{\mathrm{cc}}-{\mathrm{n̅}}_{e}\right)+\frac{4\pi e_{au}}{k_{B}T}\left(q_{c}{\mathrm{n̅}}_{c}-q_{a}{\mathrm{n̅}}_{a}\right)$$

The dimensionless boundary conditions in the bulk solution, *x̅* = ±(*L*_S_ + *L*_M_), naturally become φ = 0 and ϕ̅ = 0.

The dimensionless electron density is written as:

n̅e=a02mekBTℏ2π∑φ2⁡ln(1+e(μ~¯e−ε)) 17

where φ=φ∫φ2⁡dx is the normalized wave function.^76^

### Constant-Potential Conditions

Here we explain how constant potential conditions are preserved in DPFT and Kohn–Sham–Poisson–Boltzmann models. For DPFT models, controlling the constant potential is realized by controlling *μ̃*_e_, namely, the electrochemical potential of electrons, in governing equations in eqs 9 and 10. For Kohn–Sham–Poisson–Boltzmann models, adjusting *μ̃*_e_ in eq 7 can also achieve constant-potential calculations.

### Numerical Flowchart of Self-Consistent Solution

In this part, we first introduce the numerical method of solving the Kohn–Sham equation and Poisson–Boltzmann equation, separately, then present the flowchart of self-consistent solutions.

#### Finite Difference Method for Solving the Kohn–Sham Equation

Following Halpern et al.,^77^ we use the finite difference method with a second-order center difference scheme to discretize the Kohn–Sham equation. For a 1D uniform mesh grid, the discretization of eq 15 leads to:

18 $$\overset{n}{\underset{j=1}{\sum }}H_{\mathrm{ij}}\varphi_{i}=\mathrm{\varepsilon ̅}\varphi_{i}$$

where the index *i* denotes the grid point and *d* *x̅* is the uniform step width on the 1D mesh. ***H***_***ij***_ is the Hamiltonian matrix:

$$H_{\mathrm{ij}}=\{\begin{array}{ll} \frac{\hbar^{2}}{m_{e}a_{0}^{2}k_{B}T}\frac{1}{d{\mathrm{x̅}}^{2}}+{\mathrm{v̅}}_{\mathrm{eff}}, & \mathrm{if},i=j\\ -\frac{1}{2}\frac{\hbar^{2}}{m_{e}a_{0}^{2}k_{B}T}\frac{1}{d{\overset{¯}{x}}^{2}}, & \mathrm{if},i=\left|j\pm 1\right|\\ 0, & \mathrm{otherwise} \end{array}$$ 19

We notice ***H***_***ij***_ is a symmetric tridiagonal matrix with the uniform mesh, which can be easily solved with numerical method. While the nonuniform mesh step is preferable for certain problems, this would destroy the symmetry of the matrix ***H***_***ij***_ and increase numerical complexities. Tan et al. used a proper matrix transformation that preserves the symmetry of the discretized Kohn–Sham equation even with a nonuniform mesh step, thereby reducing the computation time.^44^

#### Finite Element Method for Solving the Poisson–Boltzmann Equation

The Poisson–Boltzmann equation shown in eq 16 can be solved using the finite difference method, finite element method, and finite volume method. Considering the stability and capability of extension to more complex cases, we use the finite element method to solve it, with details provided in supplementary note 6 in the Supporting Information.

Since the Poisson–Boltzmann equation is nonlinear, an iteration strategy is needed. We define a residual vector *R*(*ϕ̅*_*i*_^*k*^), which is expected to be zero,

20 $$R\left({\mathrm{\phi ̅}}_{i}^{k}\right)=K_{ij}\left({\mathrm{\phi ̅}}_{i}^{k}\right)c\left({\mathrm{\phi ̅}}_{i}^{k}\right)-F\left({\mathrm{\phi ̅}}_{i}^{k}\right)=0$$

where the superscript *k* denotes the *k* ^th^ iteration, *K*_*ij*_(*ϕ̅*_*i*_^*k*^) is the stiffness matrix, *F*(*ϕ̅*_*i*_^*k*^) is the source vector, and *c*(*ϕ̅*_*i*_^*k*^) are coefficients to be determined.

Using the first-order Taylor expansion, we obtain

$$R\left({\mathrm{\phi ̅}}_{i}^{k+1}\right)\approx R\left({\mathrm{\phi ̅}}_{i}^{k}\right)+\frac{\partial R\left({\mathrm{\phi ̅}}_{i}^{k}\right)}{\partial {\mathrm{\phi ̅}}_{i}^{k}}{|}_{{\mathrm{\phi ̅}}_{i}={\mathrm{\phi ̅}}_{i}^{k}}\cdot \delta {\mathrm{\phi ̅}}_{i}^{k}=0$$ 21

with δϕ̅_*i*_^*k*^ being the changing value between *k* ^th+1^ and *k* ^th^ iteration.

Therefore, we obtain

$$\frac{\partial R\left({\mathrm{\phi ̅}}_{i}^{k}\right)}{\partial {\mathrm{\phi ̅}}_{i}^{k}}{|}_{{\mathrm{\phi ̅}}_{i}={\mathrm{\phi ̅}}_{i}^{k}}\cdot \delta {\mathrm{\phi ̅}}_{i}^{k}=-R\left({\mathrm{\phi ̅}}_{i}^{k}\right)$$ 22

Equation 21 is rewritten as

23 $$\delta {\mathrm{\phi ̅}}_{i}^{k}=J\backslash \left(-R\left({\mathrm{\phi ̅}}_{i}^{k}\right)\right)$$

where $J=\frac{\partial R\left({\mathrm{\phi ̅}}_{i}^{k}\right)}{\partial {\mathrm{\phi ̅}}_{i}^{k}}{|}_{{\mathrm{\phi ̅}}_{i}={\mathrm{\phi ̅}}_{i}^{k}}$ is the so-called Jacobian matrix.^78^

In the *k* ^th^+1 iteration, the updated ϕ̅_*i*_^*k*+1^ = ϕ̅_*i*_^*k*^ + δϕ̅_*i*_^*k*^, which is referred to as the Newton–Raphson iteration.^79^ Repeating iteration until ϕ̅_*i*_^*k*^ is unchanged, we can obtain convergent solutions of ϕ̅_*i*_.

Notably, the boundary conditions will modify the stiffness matrix and source term, see more details in supplementary note 7 in the Supporting Information.

#### Flowchart for Self-Consistently Solving the Kohn–Sham–Poisson–Boltzmann Equations

A self-consistent method is developed to solve the coupled Kohn–Sham–Poisson–Boltzmann equations,^64^ as shown in Figure 2. Initial guesses of ϕ̅_0_ and *n̅*_e_^0^ used are detailed in supplementary note 8 in the Supporting Information. Using the finite difference method with a dimensionless grid step of 0.04 to solve the Kohn–Sham equation, we obtain the eigenvalues and eigenvectors, namely, the distributions of φ_*i*_ and ε̅, respectively. Thereafter, we calculate an updated electron density *n̅*_e_^*m*^ using eq 17, with the superscript *m* denoting the *m* ^th^ iteration. Subsequently, the updated electron density is used to solve the (modified) Poisson–Boltzmann equation to obtain a new electrostatic potential ϕ̅_*m*_. This step is carried out using the finite element method combined with the Newton–Raphson iteration, which ensures high numerical accuracy and stability when dealing with nonlinear terms. The newly computed potential ϕ̅_*m*+1_ and electron density *n̅*_e_^*m*+1^ are compared to their previous values to evaluate the convergence tolerance, defined as |ϕ̅_*m*+1_ – ϕ̅_*m*_|&|*n̅*_e_^*m*+1^ – *n̅*_e_^*m*^|, which is required to be smaller than 10^–6^. If both quantities are smaller than this threshold, the self-consistent field cycle is considered to be converged, and the results are reported. Otherwise, we repeat the iteration with updated ϕ̅_*m*_ and *n̅*_e_^*m*^ until the errors are smaller than the predefined values. To evaluate the influence of mixing parameters, Figure 3(f) further compares convergence performance obtained with linear mixing parameters 0.05 and 0.5. For the DPFT models described using the TFvW and Pauli–Gaussian functionals, a denser mesh with a step of 0.02 at the metal–solution interface is required to accurately capture the large gradients.

**Figure 2:**
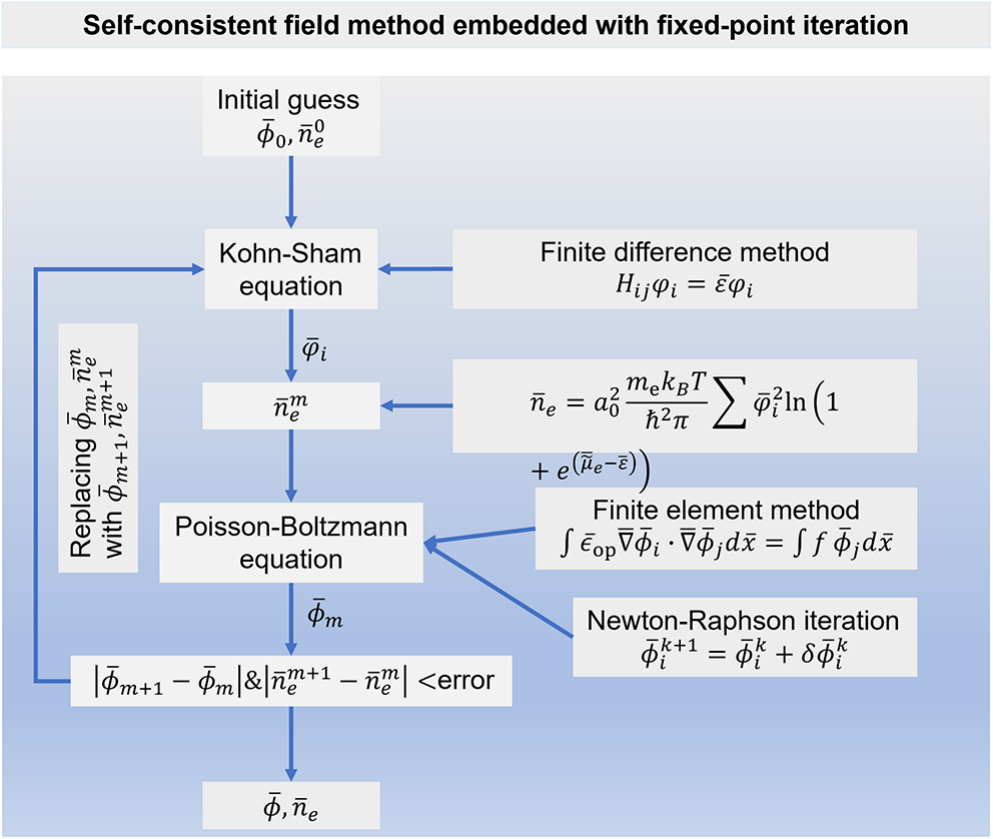
Flowchart for self-consistently solving the coupled Kohn–Sham–Poisson–Boltzmann equations.

**Figure 3:**
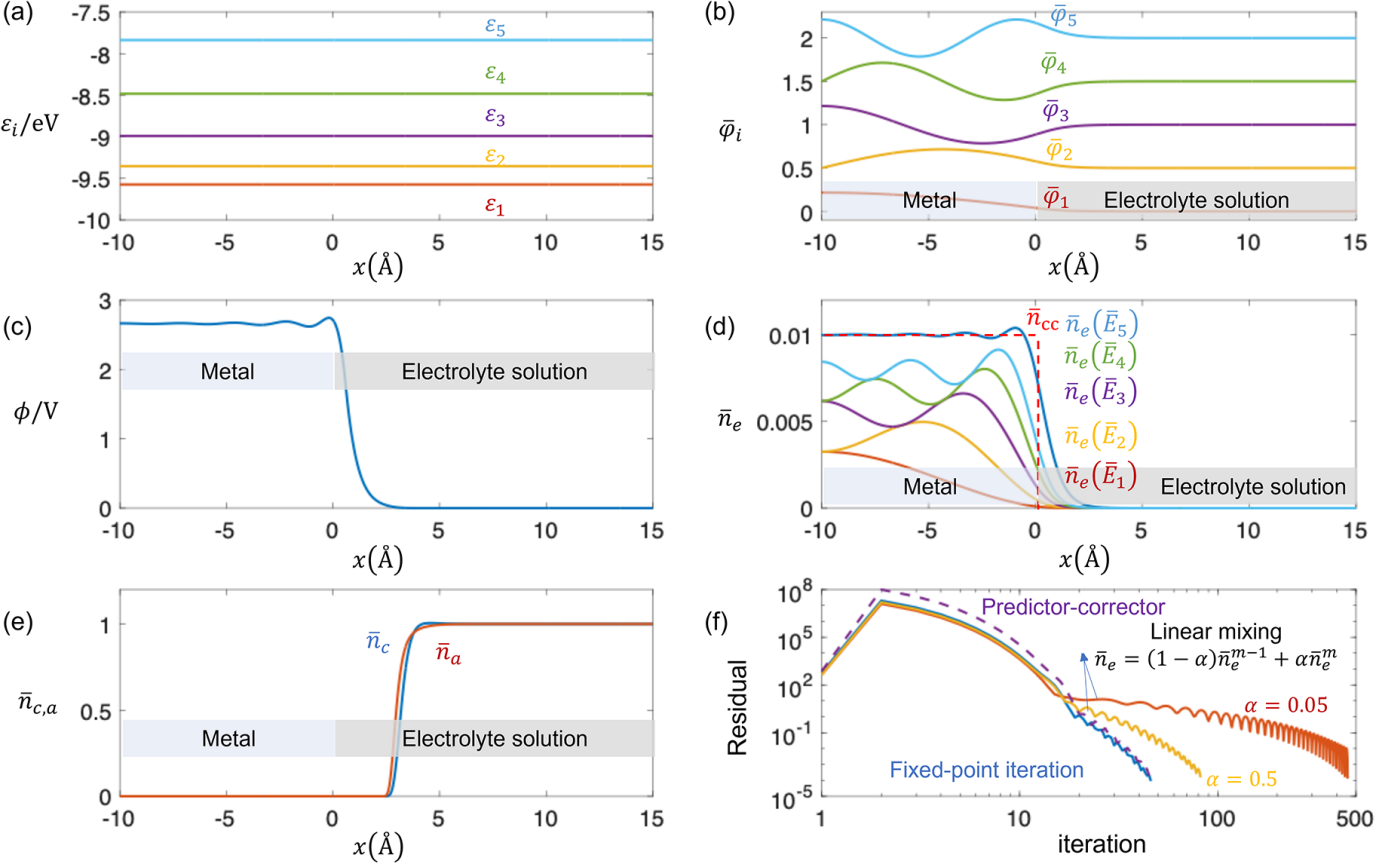
Basic results of the Kohn–Sham–Poisson–Boltzmann model at *μ̃*_e_ = −3.6 eV. (a) Disbributions of the first five energy levels ε_*i*_ of electrons, (b) distributions of the first five normalized wave functions φ̅_*i*_, (c) distribution of electrostatic potential ϕ, (d) distributions of electron density and subband’s contributions, (e) distributions of dimensionless cation *n̅*_c_ and anion density *n̅*_a_, (f) comparison of residuals among the fixed-point iteration, linear mixing method, and predictor–corrector method. Parameters used in calcualtion are as follows: *n̅*_cc_ = 0.01, *n*_c,a_^bulk^ = 1 M (number density is concentration times Avogadro constant throughout this paper), *L*_M_ = 20, *L*_S_ = 30, *d*_c,a_ = 4*a*_0_, *D*_*l*_ = 0.5/6 eV, and β_c,a_ = 1.

### Model Parameters

We divide the parameters into three groups: structural parameters including *L*_M_ and *L*_S_, materials properties including *n*_cc_, *n*_c,a_^bulk^, *a* and ϵ_op_, and empirical parameters *d*_c,a_, *D*_*l*_, β_c,a_, θ_T_ and μ_PG_. Table 1 gives the model parameters for the base case.

**Table 1:** Model Parameters for the Base Case

symbol	item	values
Structural parameters		
*L*_M_	Dimensionless metal thickness	20
*L*_S_	Dimensionless electrolyte solution thickness	30
Material properties		
*n*_cc_	Cationic core density	0.01*a*_0_^–3^
*n*_c,a_^bulk^	Bulk ions density	1 M
*a*	Ion size	0 (neglecting ions size)
ϵ_op_	Dielectric permittivity	ϵ_0_
Empirical parameters		
*d*_c,a_	Equilibrium distance of cation/anion to the metal surface	4*a*_0_
*D*_*l*_	Depth of the Morse potential well	0.5/6 eV
β_c,a_	Coefficient controlling the width of the Morse potential well	1
θ_*T*_	Empirical parameter in TFvW energy functional	Calibrated to Kohn–Sham–Poisson–Boltzmann models
μ_PG_	Empirical parameter in Pauli–Gaussian energy functional	

For the structural parameters, *L*_M_ and *L*_S_ denote dimensionless thicknesses of the metal and electrolyte solution ranging from 1 nm to tens of nanometers in typical DFT calculations.^80^ Here we set *L*_M_ = 20 and *L*_S_ = 30 referenced to *a*_0_ (0.529 Å).

For materials properties, *n*_cc_ denotes the density of cationic cores in the metal bulk which can be estimated from the effective Wigner-Seitz radius or the cubic cell structure.^81^ The dimensionless *n̅*_cc_ with respect to *a*_0_^–3^ ranges from 0.001 to 0.01 for common metals (see supplementary note 9 in the Supporting Information.) *n*_c,a_^bulk^ is the cation/anion density in the solution bulk that ranges from 1 mM to 1 M.^1^ The ion size *a* ranges from 1 Å to 5 Å.^82^ ϵ_op_ denotes the permittivity which is different between metal and electrolyte solution. For typical metals, ϵ_op_ is in the range from 2 ϵ_0_ to 9 ϵ_0_.^33^ While for the electrolyte solution, ϵ_op_ ranges from 5 ϵ_0_ to 20 ϵ_0_ (organic solvent)^83^ to 78.5 ϵ_0_ (aqueous solvent).^1,2^ Here we set *n*_*c*,*a*_^bulk^ = 1 M, *a* = 0 and ϵ_op_ = ϵ_0_, which means neglecting the ion size and assuming ϵ takes the vacuum’s value without spatial distributions.

For the empirical parameters, *d*_c,a_ is the equilibrium distance of cation/anion to the metal surface, which ranges from 2 Å to 10 Å.^34,81^ *D*_*l*_ is the depth of the Morse potential well that ranges from 0.01 to 0.1 eV.^34,81^ β_c,a_ is the coefficient that controls the width of the potential well to be adjusted for a good agreement with experiments. θ_T_ and μ_PG_ are empirical parameters to be determined by first-principle calculations or experiments.^34^ We set *d*_c,a_ = 4*a*_0_, *D*_*l*_ = 0.5/6 eV, β_c,a_ = 1, while θ_T_ and μ_PG_ are calibrated by the solutions of the Kohn–Sham–Poisson–Boltzmann model.

## Results and Discussion

First, we present results of the Kohn–Sham-Poisson–Boltzmann model with basic setups, including the distributions of energy levels, wave functions, electrostatic potential, electron density, and comparison of convergence plots using different iteration schemes. Second, we compare the electrostatic potential and electron density calculated from the Kohn–Sham–Poisson–Boltzmann and DPFT models using the TFvW and Pauli–Gaussian functionals, respectively. Third, we compare the *C*_dl_ values calculated from the Kohn–Sham–Poisson–Boltzmann and DPFT models using the TFvW and Pauli–Gaussian functionals, respectively. Fourthly, we compare the DPFT model and Kohn–Sham–Poisson–Boltzmann model using Perdew–Burke–Ernzerhof (PBE) functional for the exchange-correlation functional. Lastly, we improve the electrolyte solution model by accounting for finite ion size via a lattice-gas model and solvent polarization through a Langevin function and compare the results calculated from the DPFT model and the Kohn–Sham-Poisson–Boltzmann model.

### A Glance at the Kohn–Sham–Poisson–Boltzmann Model

Following the procedure shown in Figure 2, we obtain numerical solutions at μ̃_e_ = – 3.6 eV, as shown in Figure 3. Only one metal–solution interface is shown here. Figure 3(a) displays the first five energy levels ε_*i*_ of electrons. The energy gap Δε_*i*_ increases at higher ε_*i*_, which can be understood using the standard “particle in a box” model because the metal bulk approximates the scenario of an infinite potential well. In the “particle in a box” model, Δε_*i*_ is proportional to 2*i* + 1.^77^

The first five normalized wave functions φ̅_*i*_ are shown in Figure 3(b). As ε_*i*_ increases, the period of φ̅_*i*_ in metal decreases and the distributions become denser. Figure 3(c) displays the distribution of electrostatic potential ϕ. It is almost constant in the metal bulk and decreases to zero within a region of a few Å in the solution bulk. Figure 3(d) shows the distributions of dimensionless electron density *n̅*_e_ and subband’s contribution *n̅*_e_ (ε_*i*_). *n̅*_e_ exhibits Friedel oscillations at the metal–solution interface, which is absent in DPFT or orbital-free DFT. Figure 3(e) displays the distributions of dimensionless cation density, *n̅*_c_, and anion density, *n̅*_a_, where minor difference is observed at the metal-solution interface. This means the system is nearly at the electroneutrality case with a controlled *μ̃*_e_ = −3.6 eV, namely, under the potential of zero charge condition.

Figure 3(f) compares the residuals among the fixed-point iteration, linear mixing method and predictor-corrector method. The convergence speeds are almost the same between the fixed-point iteration and predictor-corrector method, whereas the linear mixing method is slower. Increasing the mixing factor α accelerates convergence as the next-step solution will be closer the convergent value. Additionally, the linear mixing method exhibits some hysteresis, which arises from the next-step solution retaining information from the previous-step solution. Therefore, hysteresis is suppressed with increasing α from 0.05 to 0.5.

### Comparison between Kohn–Sham–Poisson–Boltzmann and DPFT Models in Terms of the Electron Density and Electrostatic Potential

We compare the electron density and electrostatic potential calculated from the Kohn–Sham–Poisson–Boltzmann and DPFT models using the TFvW and Pauli–Gaussian kinetic energy functionals. The comparison is conducted at *μ̃*_e_ = −3.57 eV, which is the potential of zero charge for the Kohn–Sham–Poisson–Boltzmann model at *n̅*_cc_ = 0.01, see supplementary note 10 in the Supporting Information.

#### DPFT with TFvW Kinetic Energy Functional

To compare the differences between the two approaches, we define two normalized variables, the normalized electron density *n*_e,n_ = *n̅*_e_/*n̅*_cc_ and the normalized electron density error Δ*n*_e,n_ = (*n̅*_e_^DPFT^ – *n̅*_e_^Kohn–Sham–Poisson–Boltzmann^)/*n̅*_cc_.

Figure 4(a) shows the distributions of Δ*n*_e,n_ with increasing θ_T_ at *E*_F_ = −3.57 eV. Three different peaks (peak 1: ∼−2.5 Å, peak 2: ∼−1 Å, peak 3: 0–1 Å) for Δ*n*_e,n_ near the metal/solution interface are observed. We notice that all three peaks change with increasing θ_*T*_. Specifically, the first two peaks are more pronounced while the third peak exhibits no clear trend with increasing θ_*T*_. Figure 4(b) shows the distributions of *n*_e,n_ with increasing θ_*T*_. We notice that the electron spillover is more pronounced with increasing θ_*T*_ due to the stronger gradient correction at the metal/solution interface (larger electron density gradient region). The TFvW kinetic functional with θ_*T*_ = 0.7 gives a better approximation in terms of *n*_e,n_. Figure 4(c) shows the comparison of the electrostatic potential calculated from the Kohn–Sham–Poisson–Boltzmann and DPFT models with increasing θ_*T*_. The surface potential drop increases as θ_*T*_ increases, which is caused by enhanced electron spillover as shown in Figure 4(b). Figure 4(d) shows the electrostatic potential calculated from the Kohn–Sham–Poisson–Boltzmann and the DPFT models in the region of the metal/solution interface, where θ_*T*_ = 0.6 gives a more accurate approximation. Comparison of Figure 4(b) and (d) shows that the optimal value of θ_*T*_ is different for *n*_e,n_ and ϕ distributions. Moreover, we observe oscillations within the metal in the electrostatic potential calculated using the Kohn–Sham–Poisson–Boltzmann model, which are absent in the DPFT model. These oscillations arise from the oscillatory distributions of the electron density shown in Figure 4(a). Since $\phi \left(x\right)=-\int_{x_{b}}^{x_{i}}\frac{1}{\epsilon_{\mathrm{op}}\left(x\right)}dx\int_{x_{b}}^{x\prime }e_{0}\left(n_{\mathrm{cc}}-n_{e}+q_{c}n_{c}-q_{a}n_{a}\right)dx$ , the oscillation of electron density will cause oscillatory distributions of ϕ­(*x*) within the metal.

**Figure 4:**
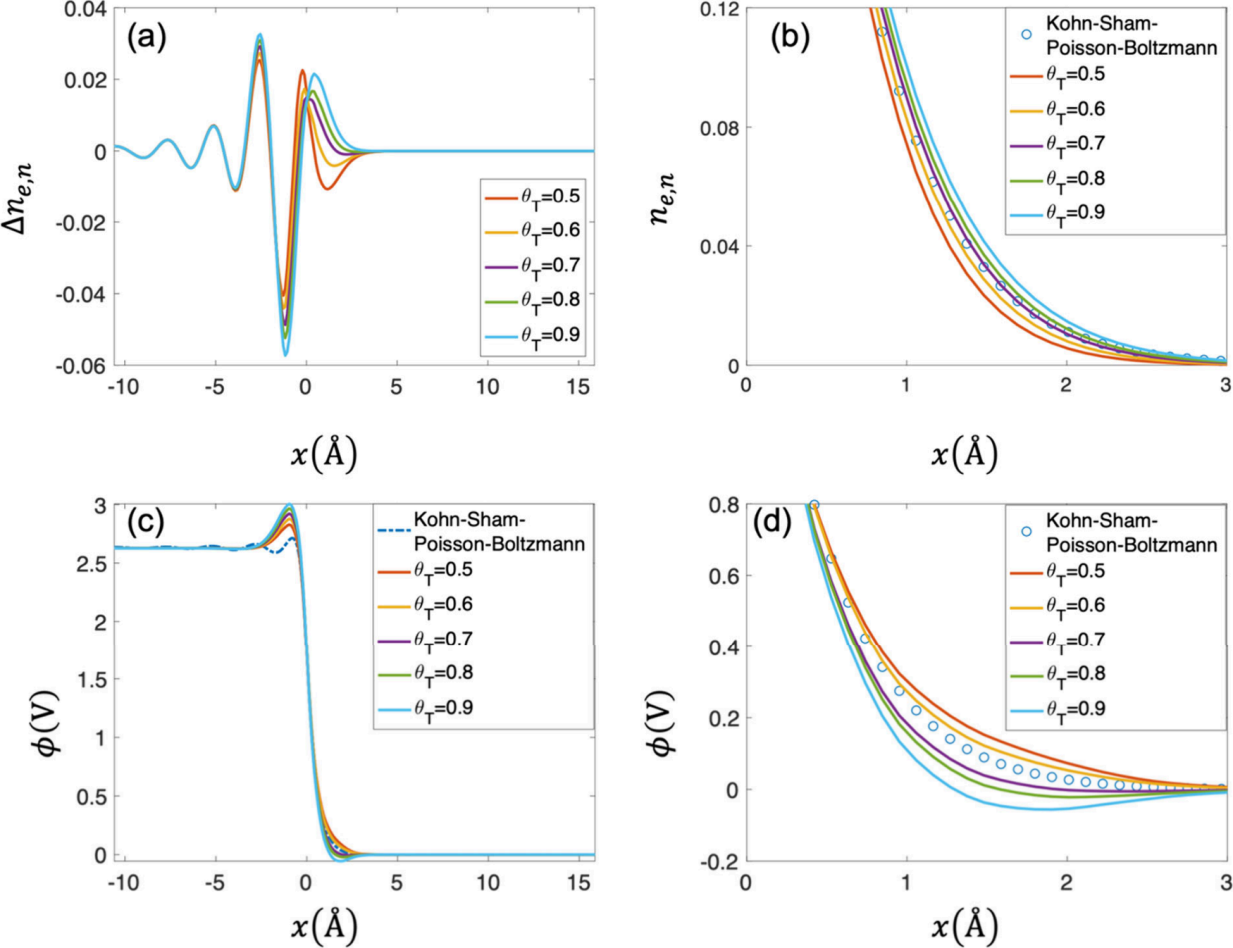
Comparison between the DPFT model with the TFvW functional and Kohn–Sham–Poisson–Boltzmann model at *μ̃*_e_ = −3.57 eV in terms of (a) normalized electron density error Δ*n*_e,n_ = (*n̅*_e_^DPFT^ – *n̅*_e_^Kohn–Sham–Poisson–Boltzmann^)/*n̅*_cc_, (b) normalized electron density *n*_e,n_ = *n̅*_e_/*n̅*_cc_, (c) electrostatic potential, (d) electrostatic potential at the region of the metal/solution interface. Parameters used in calculation are the same as those in Figure 3.

#### DPFT with Pauli–Gaussian Kinetic Energy Functional

Figure 5(a) shows the distributions of Δ*n*_e,n_ with increasing μ_PG_ at a fixed θ_*T*_ = 5/3, a default value in the Pauli–Gaussian functional.^39^ Three different peaks (peak 1: ∼−2.5 Å, peak 2: ∼−1 Å, peak 3: 0–1 Å) for Δ*n*_e,n_ near the metal/solution interface are also observed. The difference from the Figure 4 (a) is that only the second and third peaks change with increasing μ_PG_ while the first peak keeps unchanged. This indicates that a large θ_*T*_ will overestimate the electron density which cannot be corrected by the *e* ^–μ_PG_ *s* ^2^^ term. For the Pauli–Gaussian functional, $\theta_{T}=\frac{5}{3}$ overestimates the electron density (peak 1), compared with that from the Kohn–Sham–Poisson–Boltzmann model. Figure 5(b) shows the distributions of *n*_e,n_ with increasing μ_PG_. We notice that the Pauli–Gaussian functional with μ_PG_ = 0.35 gives a more accurate approximation in terms of *n*_e,n_. Figure 5(c) shows the comparison of the electrostatic potential calculated from the Kohn–Sham–Poisson–Boltzmann and DPFT models with increasing μ_PG_. The surface potential drop decreases as μ_PG_ increases, which is consistent with the understanding that a larger μ_PG_ suppresses the electron spillover shown in Figure 5(b). Figure 5(d) shows the electrostatic potential calculated from the Kohn–Sham–Poisson–Boltzmann and DPFT models in the region of the metal–solution interface, where μ_PG_ = 0.25 gives a more accurate approximation in terms of the distribution of the electrostatic potential. Figure 5(b) and (d) also suggest different optimal values of μ_PG_ for *n*_e,n_ and ϕ. The Kohn–Sham–Poisson–Boltzmann model exhibits oscillations in the electrostatic potential within the metal calculated, which are absent in the DPFT model with the Pauli–Gaussian kinetic energy functional. These oscillations also arise from the oscillatory distributions of the electron density shown in Figure 5(a).

**Figure 5:**
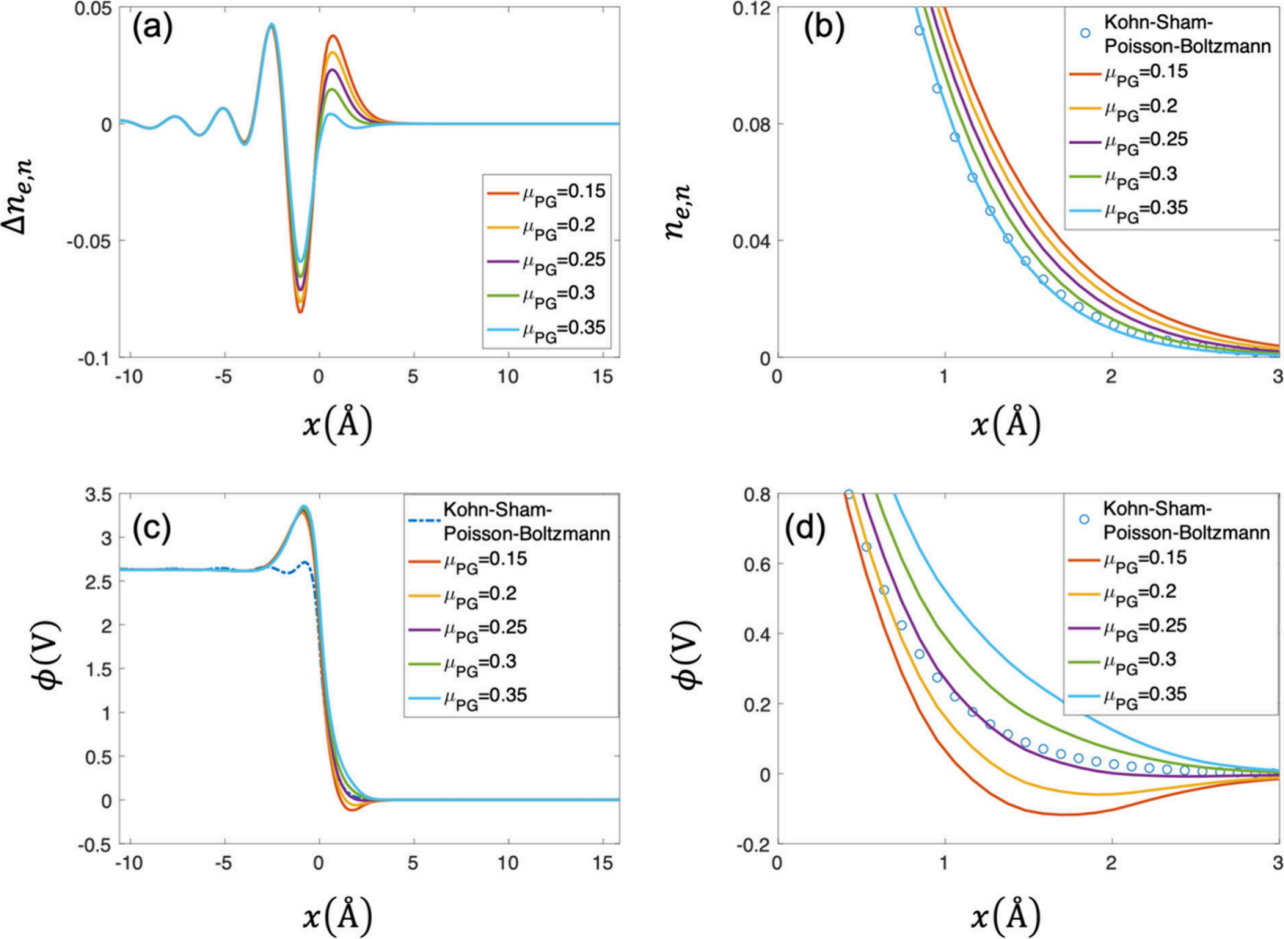
Comparison between the DPFT model with the Pauli-Gaussian functional and Kohn–Sham–Poisson–Boltzmann model at *μ̃*_e_ = −3.57 eV in terms of (a) normalized electron density error Δ*n*_e,n_ = (*n̅*_e_^DPFT^ – *n̅*_e_^Kohn–Sham–Poisson–Boltzmann^)/*n̅*_cc_, (b) normalized electron density *n*_e,n_ = *n̅*_e_/*n̅*_cc_, (c) electrostatic potential, (d) electrostatic potential at the region of the metal/solution interface. Parameters used in calculation are the same as those in Figure 3.

### Comparison between Kohn–Sham–Poisson–Boltzmann and DPFT Models in Terms of Double-Layer Capacitance Curves

In this part, we explain the calculation of the surface free charge and double-layer capacitance, and study the effect of θ_*T*_ and μ_PG_ and the charge density of metal cationic cores on the double-layer capacitance.

#### Definitions of the Surface Free Charge and Double-Layer Capacitance

The surface free charge density for the EDL is defined as:^33^

24 $$\sigma_{\mathrm{free}}=\int_{0}^{+\infty }{(n}_{\mathrm{cc}}-n_{e})e_{0}dx=\frac{e_{0}}{a_{0}^{2}}\int_{0}^{+\infty }\left({\mathrm{n̅}}_{\mathrm{cc}}-{\mathrm{n̅}}_{e}\right)d\mathrm{x̅}$$

where the second equal sign is due to the dimensionless definition.

Double layer capacitance (*C*_dl_) curve can be obtained by differentiating σ_free_ with electrochemical potential of electrons μ̃_e_, namely,

25 $$C_{\mathrm{dl}}=-e_{0}\frac{\partial \sigma_{\mathrm{free}}}{\partial {\mathrm{\mu ̃}}_{e}}$$

*C*_dl_ exhibits a minimum extremum at the potential of zero charge condition in dilute solutions,^72^ while a maximum extremum in highly concentrated solutions.^9,33^

#### Effect of θ_*T*_ and μ_PG_

Figure 6(a) shows the comparison of *C*_dl_ calculated from Kohn–Sham–Poisson–Boltzmann and DPFT models with the TFvW functional. From the *C*_dl_ curve calculated by the Kohn–Sham-Poisson–Boltzmann model, we determine the potential of zero charge to be approximately −3.6 eV, which is close to −3.57 eV calculated from the surface free charge (see supplementary note 5 in the Supporting Information). For the DPFT model described using the TFvW functional, the potential of zero charge becomes more negative with increasing θ_*T*_. We calibrate θ_*T*_ = 0.7 in the DPFT model to capture the potential of zero charge calculated by the Kohn–Sham–Poisson–Boltzmann model. The DPFT model with θ_*T*_ = 0.7 also captures well the electron spillover in Figure 4(b). However, *C*_dl_ is overrated at less negative μ̃_e_, shown in Figure 6(a), which results from the overestimation of the electron spillover.

**Figure 6:**
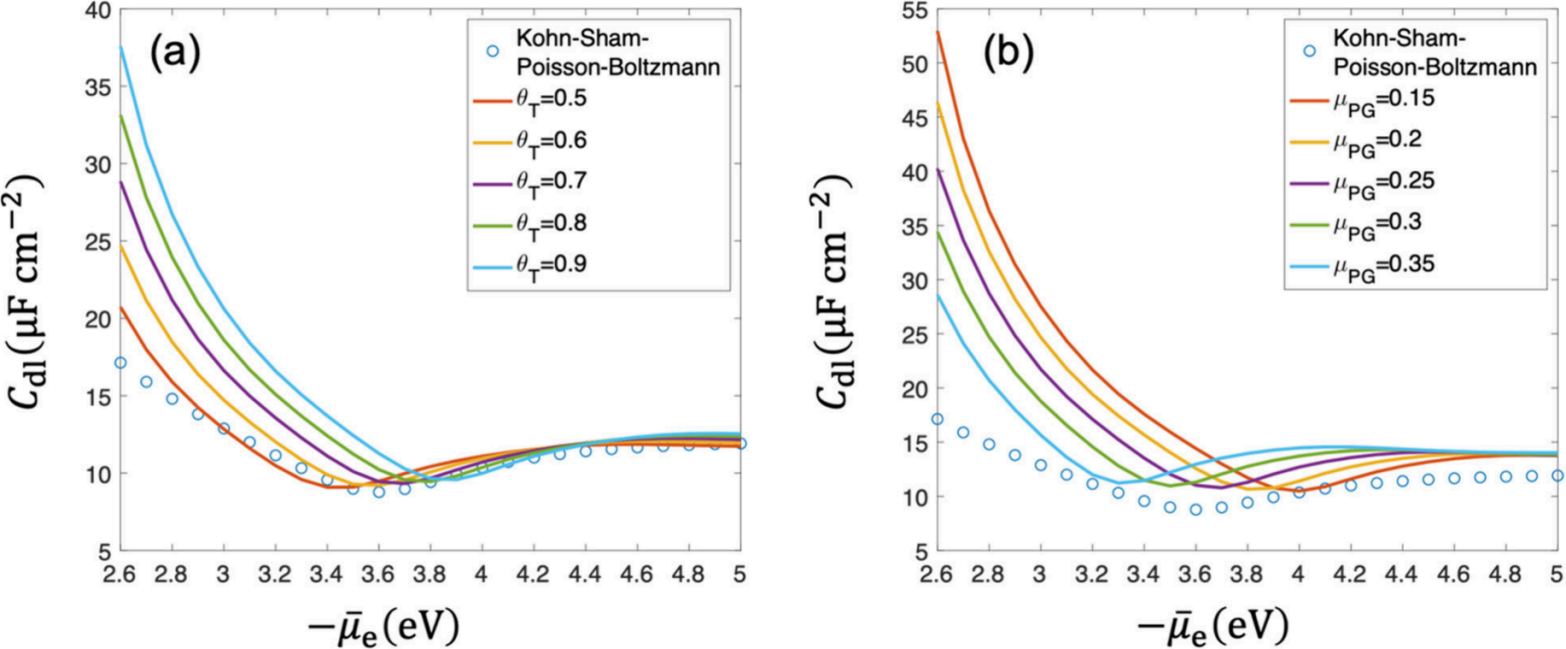
Comparison between DPFT model using (a) TFvW functional, (b) Pauli–Gaussian functional and Kohn–Sham–Poisson–Boltzmann model in terms of double-layer capacitance at *n̅*_cc_ = 0.01. Parameters used in the calculation are the same as those in Figure 3.

The DPFT model described using the Pauli–Gaussian functional exhibits poorer performance in predicting *C*_dl_ shown in Figure 6(b), regardless of whether the system is positively or negatively charged. Especially at less negative μ̃_e_, a large *C*_dl_ arises from the larger electron density error, though it predicts the potential of zero charge better at μ_PG_ = 0.25 in accordance with Figure 5(d).

To quantify the differences between the DPFT and Kohn–Sham–Poisson–Boltzmann models, we perform a comprehensive error analysis (detailed results are provided in supplementary note 11 in the Supporting Information). We notice that θ_*T*_ = 0.7 and μ_PG_ = 0.25 are optimized parameters in terms of μ̃_e_^pzc^, whereas the DPFT models at θ_*T*_ = 0.5 and μ_PG_ = 0.35 yield the minimum mean error in terms of *C*_dl_.

#### Effect of the Charge Density of Metal Cationic Cores

Metal cationic core density *n̅*_cc_ is a characteristic parameter of metals in our model. As shown in Figure 6 and Figure 7, the *C*_dl_ curve changes at a smaller *n̅*_cc_ in both Kohn–Sham–Poisson–Boltzmann and DPFT models. Figure 7(a) and (b) show that the overrated *C*_dl_ calculated by the DPFT models is suppressed at less negative μ̃_e_ as *n̅*_cc_ decreases. This is because that the overestimation of the electron spillover is suppressed at a smaller *n̅*_cc_. The potential of zero charge determined by the Kohn–Sham–Poisson–Boltzmann model shifts from ∼−3.6 eV to −3.3 eV as *n̅*_cc_ decreases from 0.01 to 0.005. Though the DPFT model described using the TFvW functional with θ_*T*_ = 0.7 still fits the potential of zero charge prediction, θ_*T*_ = 0.6 fits the *C*_dl_ best. The Pauli–Gaussian functional still exhibits a poorer performance than the TFvW functional and predicts the potential of zero charge value best at μ_PG_ = 0.3. A quantitative error analysis is detailed in supplementary note 12 in the Supporting Information. We notice that θ_*T*_ = 0.7 and 0.8, as well as μ_PG_ = 0.2 are optimized parameters in terms of μ̃_e_^pzc^, whereas the DPFT models at θ_*T*_ = 0.5 and μ_PG_ = 0.35 have the minimum mean error in terms of *C*_dl_. From the comparison of *C*_dl_, we conclude that the TFvW functional provides a more accurate description of metal-solution interfaces than the Pauli–Gaussian functional, though the latter is reported to perform better in bulk metals and semiconductors.^39^ This highlights the importance of examining orbital-free DFT in the context of metal–solution interfaces.

**Figure 7:**
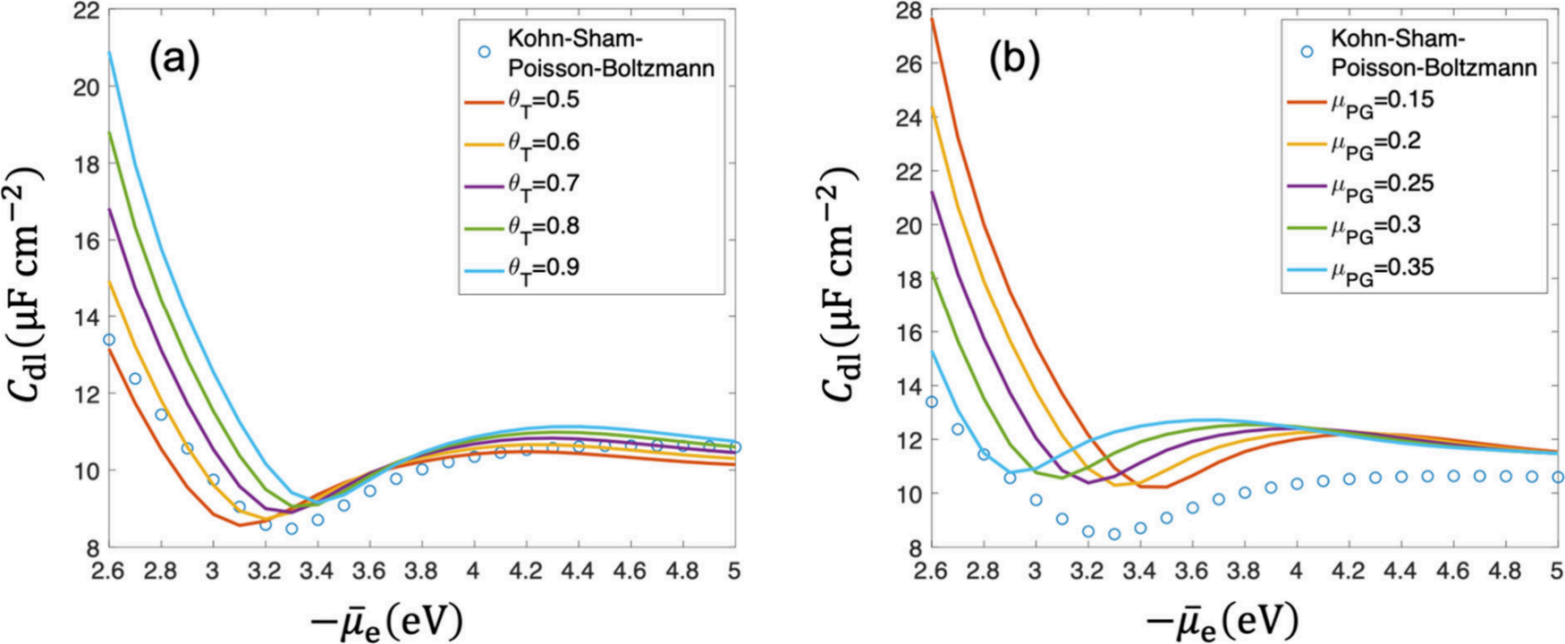
Comparison between DPFT model with (a) TFvW functional and (b) Pauli–Gaussian functional and Kohn–Sham–Poisson–Boltzmann model results in terms of double-layer capacitance at *n̅*_cc_ = 0.005. Other parameters used in the calculation are the same as those in Figure 3.

#### Effect of Exchange-Correlation Functional

We further compare the results calculated from the DPFT and Kohn–Sham–Poisson–Boltzmann models using an improved description of XC potential based on a general gradient approximation, namely, the Perdew–Burke–Ernzerhof (PBE) functional.

The exchange-correlation potential energy described by the PBE functional is defined as

26 $$U_{\mathrm{XC}}=\int d^{3}rv_{\mathrm{XC}}^{\mathrm{PBE}}$$

where *v*_XC_^PBE^ is the volumetric exchange-correlation energy including an exchange part and a correlation part:^84^

27 $$v_{\mathrm{XC}}^{\mathrm{PBE}}=v_{X}+v_{C}$$

where *v*_X_ is expanded as

28 $$v_{X}=e_{au}a_{0}^{-3}v_{X}^{0}\left(1+\theta_{X}s^{2}\right)$$

with the volumetric exchange energy of a uniform electron gas: $v_{X}^{0}=-\frac{3}{4}{\left(\frac{3}{\pi }\right)}^{1/3}{\left(n_{e}a_{0}^{3}\right)}^{4/3}$ . θ_X_ is a gradient coefficient tuning the contribution of the gradient term in the exchange energy. Similarly, *v*_C_ is expanded as,

29 $$v_{C}=e_{au}a_{0}^{-3}\left(v_{C}^{0}+\theta_{C}n_{e}a_{0}^{3}t^{2}\right)$$

where $t=\frac{a_{0}^{4}\left|\nabla n_{e}\right|}{4{\left(\frac{3}{\pi }\right)}^{1/6}{\left(n_{e}a_{0}^{3}\right)}^{7/6}}$ is a reduced density gradient and *v*_C_^0^ is the volumetric correlation energy of a uniform electron gas, for which we use the interpolation relation of Perdew et al.,^84^

30 $$u_{C}^{0}=-2\alpha_{1}n_{e}a_{0}^{3}\left(1+\alpha_{2}r_{s}\right)\ln\left(1+\frac{1}{\xi }\right)$$

31 $$\xi =2\alpha_{1}\left(\alpha_{3}r_{S}^{1/2}+\alpha_{4}r_{S}+\alpha_{5}r_{S}^{3/2}+\alpha_{6}r_{S}^{2}\right)$$

with α_1_ = 0.0310907, α_2_ = 0.21370, α_3_ = 7.5957, α_4_ = 3.5876, α_5_ = 1.6382, and α_6_ = 0.49294. θ_*C*_ is a gradient coefficient in the correlation energy.

Replacing the Dirac–Wigner exchange-correlation functional with the PBE functional, we calculated the *C*_dl_ from the DPFT model and Kohn–Sham–Poisson–Boltzmann model, respectively.

Figure 8(a) and (b) compares *C*_dl_ calculated from Kohn–Sham–Poisson–Boltzmann and DPFT models with the TFvW functional and Pauli–Gaussian functional at *n̅*_cc_ = 0.01, respectively. For the DPFT model with the TFvW functional, θ_*T*_ = 0.6 is a good approximation. The mean error of *C*_dl_ defined as $\mathrm{Error}=\frac{1}{N_{\mathrm{tot}}}\Sigma \frac{\left|C_{\mathrm{dl}}^{\mathrm{DPFT}}-C_{\mathrm{dl}}^{\mathrm{Kohn-Sham-Poisson-Boltzmann}}\right|}{C_{\mathrm{dl}}^{\mathrm{Kohn-Sham-Poisson-Boltzmann}}}$ with *N*_tot_ being total number of *C*_dl_^DPFT^ data points, shown in Figure 8(e), exhibits a minimum value at θ_*T*_ = 0.6, in agreement with the trend observed in Figure 8(a). The DPFT model with the Pauli–Gaussian functional exhibits poorer performance in predicting *C*_dl_, as shown in Figure 8(b), though it predicts the PZC better at μ_PG_ = 0.25, as shown in Figure 8(d). The mean error of *C*_dl_ shown in Figure 8(f) decreases with increasing μ_PG_, in accordance with Figure 8(b).

**Figure 8:**
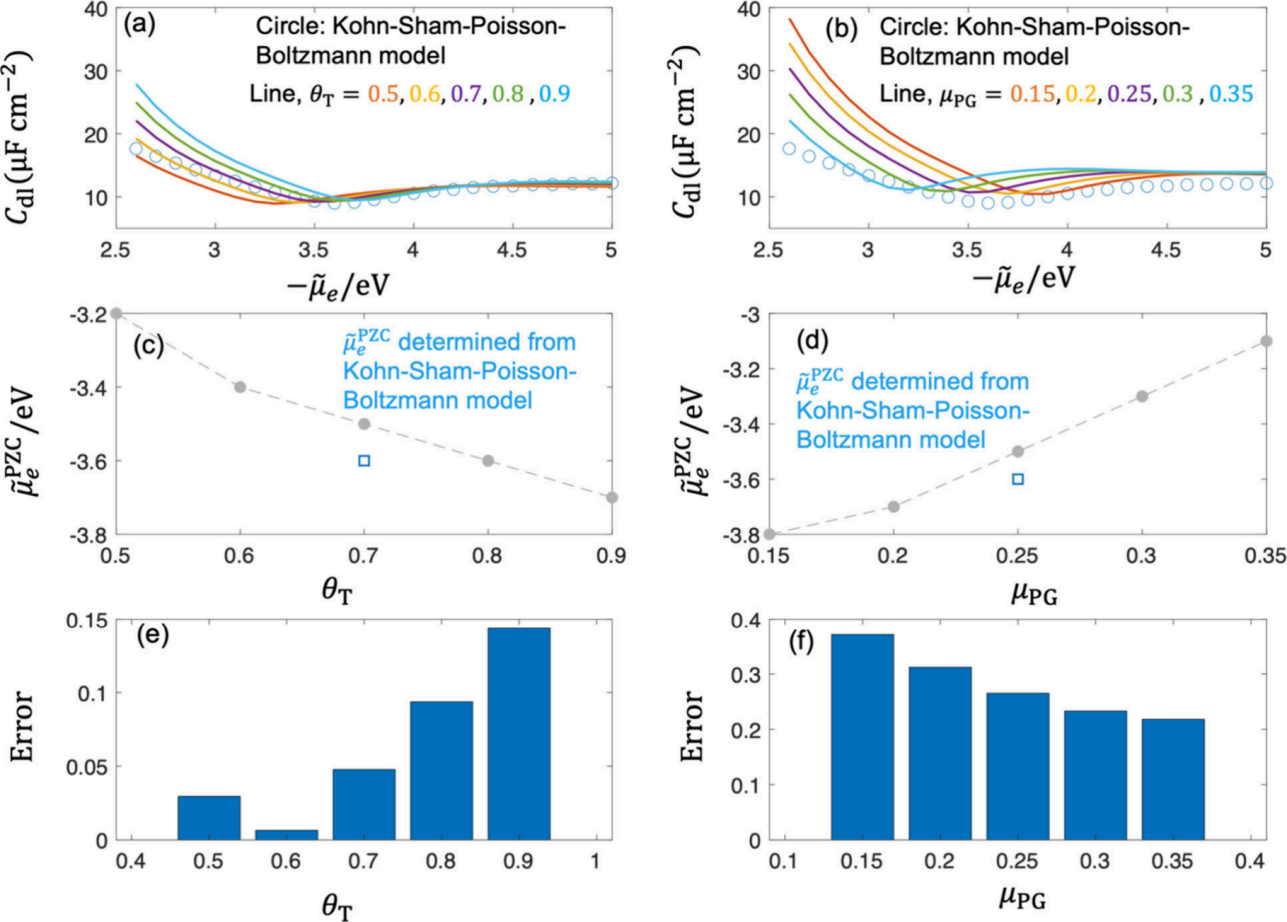
Comparison between DPFT model with TFvW functional and Pauli–Gaussian functional and Kohn–Sham–Poisson–Boltzmann model, in terms of double-layer capacitance (a, b), μ̃_e_^PZC^ (c, d), and mean error (e, f), respectively. *n̅*_cc_ = 0.01, other parameters are the same as those in Figure 3. The exchange-correlation potential is described using the PBE functional.

We note that the calibrated parameters depend on the choice of the XC potential, with the minimum error at θ_T_ = 0.5 for the Dirac-Wigner functional and at θ_T_ = 0.6 for the PBE functional, whereas the theoretical framework and benchmarking workflow are general.

#### Effect of Finite Ion Size

The finite ion size and solvent polarization have been known to importantly influence the EDL, which have been neglected in the foregoing comparison. Next, we describe the electrolyte solution more accurately by accounting for finite ion size via a lattice-gas model and solvent polarization through a Langevin function, as shown in eq S19, and perform a comprehensive comparison between the Kohn–Sham–Poisson–Boltzmann and DPFT models using the TFvW kinetic energy functional. In both models, the exchange-correlation potential is described using the PBE functional.

Figure 9(a)–(f) show the comparison of the electrostatic potential, normalized electron density, cation density, anion density, solvent density, and relative dielectric permittivity calculated from the Kohn–Sham–Poisson–Boltzmann, and DPFT models with increasing θ_*T*_, respectively. Relative to the earlier results in Figure 3, where ion size and solvent effects were neglected, the present results show pronounced maxima in the cation and anion densities, and the spatial variation of the dielectric permittivity closely follows that of the solvent density. For electrostatic potential and electron density, θ_*T*_ = 0.3 gives a more accurate approximation inside the metal, whereas θ_*T*_ = 0.5 performs better outside the metal. For cation and anion densities, θ_*T*_ = 0.4 gives more accurate approximations. A similar trend is observed for the solvent density and relative permittivity, for which θ_*T*_ = 0.4 also performs better. Figure 9(g) shows the surface charge with increasing θ_*T*_. For negatively charged surfaces, θ_*T*_ = 0.3 and θ_*T*_ = 0.4 give more accurate approximations, whereas θ_*T*_ = 0.5 performs better for positively charged surfaces. Figure 9(h) presents the comparison of *C*_dl_ with increasing θ_*T*_, where the DPFT results are overall slightly larger than those obtained from the Kohn–Sham–Poisson–Boltzmann model. The *C*_dl_ exhibits a camel shape compared with earlier results in Figure 6 and Figure 7, which is caused by the effect of finite ion size.^9^ Figure 9(i) compares μ̃_e_^pzc^, determined from DPFT and the Kohn–Sham–Poisson–Boltzmann models, showing that θ_*T*_ = 0.4 gives a more accurate approximation.

**Figure 9:**
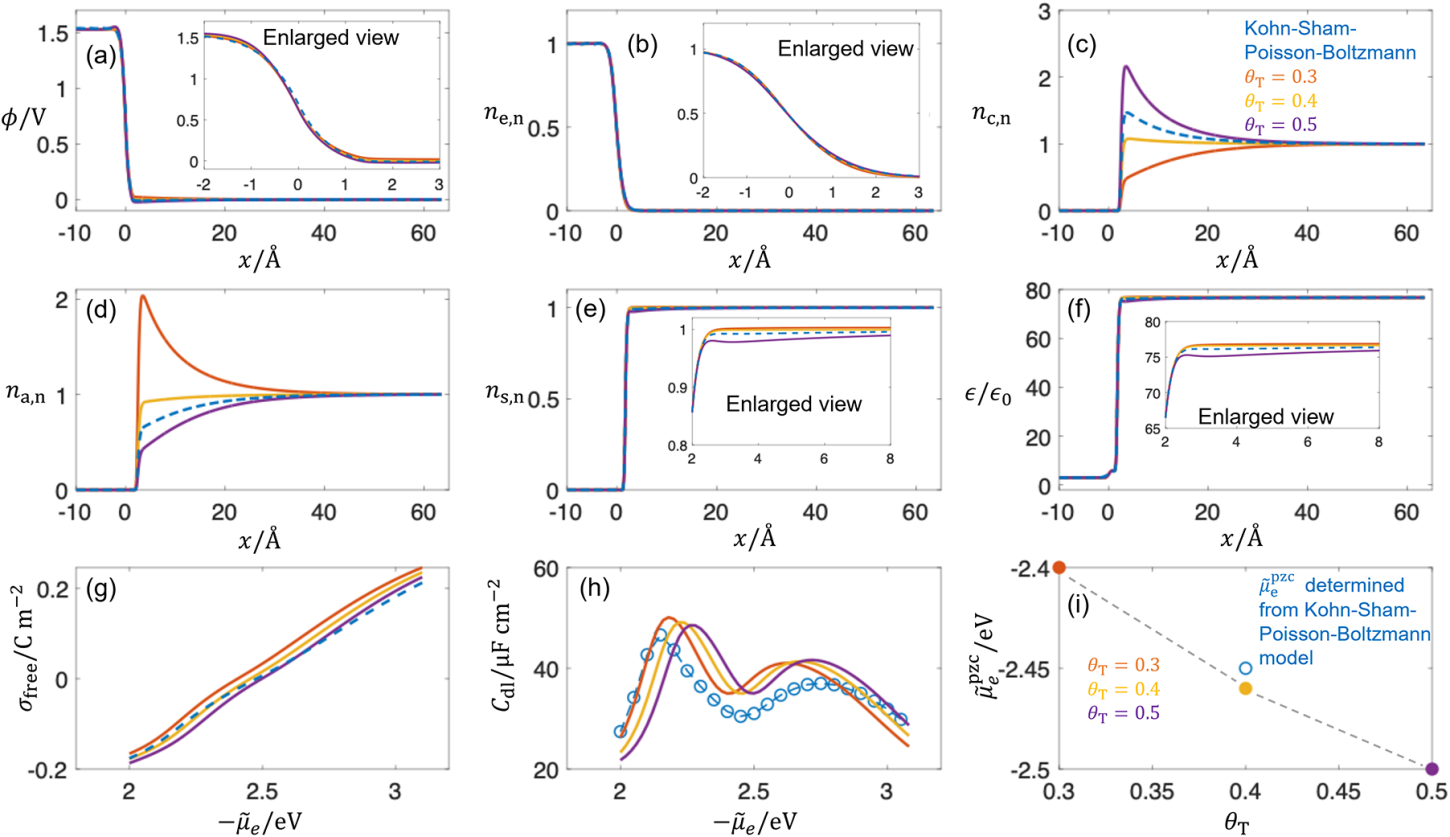
Comparison between the DPFT model with the TFvW functional and Kohn–Sham–Poisson–Boltzmann model at μ̃_e_ = −2.45 eV in terms of (a) electrostatic potential ϕ, (b) normalized electron density *n*_e,n_, (c) normalized cation density *n*_c,n_, (d) normalized anion density *n*_a,n_, (e) normalized solvent density *n*_s,n_, (f) relative dielectric permittivity ϵ/ϵ_0_, (g) surface free charge σ_free_, (h) double-layer capacitance *C*_dl_, and (i) potential of zero charge μ̃_e_^pzc^. Parameters used in the calculation are as follows: *n̅*_cc_ = 0.01, *r*_s_ = 2.75 Å, *r*_a_ = 2.7 Å, *r*_c_ = 3.6 Å, *n*_c,a_^bulk^ = 0.1 M, *n*_s_^bulk^ = 55.6 M, *p*_s_ = 1.8 D, $\epsilon_{\mathrm{op}}=\epsilon_{\mathrm{op}}^{M}+\frac{\left(\epsilon_{\mathrm{op}}^{S}-\epsilon_{\mathrm{op}}^{M}\right)}{2}\mathrm{erfc}\left(-\beta_{\mathrm{op}}\left(\left|X\right|-L_{M}\right)\right)$ , ϵ_op_^M^ = 3ϵ_0_, ϵ_op_^*S*^ = 6ϵ_0_, *D*_s_ = 0.5 eV, *d*_s_ = 2*a*_0_, β_s_ = 1, *L*_M_ = 20, *L*_S_ = 120, other parameters are the same as those in Figure 3. The exchange-correlation potential is described using the PBE functional.

## Conclusions

In this article, we have presented a Kohn–Sham–Poisson–Boltzmann theory to simulate one-dimensional metal–solution interfaces under constant-potential conditions and use it as a benchmark for orbital-free density-potential functional theory (DPFT). The Kohn–Sham–Poisson–Boltzmann model exhibits Friedel oscillations of electron density which are absent in the current DPFT model. Empirical parameters used in the DPFT models are calibrated with the solutions of the Kohn–Sham–Poisson–Boltzmann model. The comparison of electron density, electrostatic potential and double-layer capacitance shows that the Thomas–Fermi–von Weizsäcker kinetic functional provides a more accurate description of metal-solution interfaces than the Pauli–Gaussian functional, in contrast with their performance for bulk materials. The double-layer capacitance is overrated at less negative electrochemical potential of electrons for both functionals, which results from the overestimation of the electron spillover. Decreasing the density of cationic cores can suppress the electron spillover at the metal surface. The calibrated empirical parameters in DPFT models depend on the choice of the XC potential, whereas the overall trends are similar. Considering the effects of finite ion size and solvent polarization changes the ion-density distributions, dielectric permittivity, the shape of *C*_dl_, and the calibrated empirical parameter in the DPFT models. It should be noted that our assumption of a laterally uniform metal/solution interface reduces the problem to one dimension, which is a standard and widely used setup for evaluating kinetic energy functionals in planar systems. Under this ideal planar geometry, the density varies only along the surface normal, allowing us to isolate and compare the intrinsic behavior of the TFvW and Pauli–Gaussian functionals without additional geometric effects. Extending such benchmarking from one-dimensional planar to two-dimensional curved surfaces or nanoscale structures would require accounting for the additional geometric complexity, including curvature-induced anisotropy in the density gradients and spatial variations parallel to the interface.

## Supplementary Material



## Notes

Formulation of 1D Jellium model, derivations of Poisson–Boltzmann equation and governing equation for electron density, derivation of the definition of electron density, theoretical formulation of boundary conditions, discretization of the Poisson–Boltzmann equation with the finite element method, numerical implementation of boundary conditions, initial guesses of ϕ̅_0_ and *n̅*_e_^0^, estimation of dimensionless cationic core density, determination of PZC, error analysis of TFvW, and Pauli–Gaussian kinetic energy functionals (PDF)
