# Supplementary material for: Benchmarking Orbital-Free Density-Potential Functional Theory of Electrified Metal-Solution Interfaces
Source: J Chem Theory Comput. 2026 Feb 16;22(5):2607–21. doi: 10.1021/acs.jctc.5c02180 (PMC12980745; doi:10.1021/acs.jctc.5c02180)
Supplement: Supplementary file 1 [file ct5c02180_si_001.pdf]

Supporting information

Benchmarking Orbital-Free Density-Potential Functional Theory of Electrified Metal-Solution Interfaces

Chenkun Li[1,2], Xiwei Wang[1,2], Michael Eikerling[1,2], Jun Huang[1,2*]

[1]Institute of Energy and Climate Research, IET-3: Theory and Computation of Energy Materials, Forschungszentrum Jülich GmbH, 52425 Jülich, Germany

[2]Faculty of Georesources and Materials Engineering, RWTH Aachen University, 52062 Aachen, Germany

**Corresponding author: ju.huang@fz-juelich.de*

Note 1: formulation of 1D Jellium model

The Jellium slab model is shown in Figure S1, where the x-direction is a finite thickness while y and z directions are infinitely large. The wave function is as follows,

$$\varphi = \varphi_x \varphi_{xz} \tag{S1}$$

For the y and z directions, the wave function is described using the plane wave with the assumption of free particles,

$$\varphi_{yz} = \sqrt{\frac{1}{L_y}} e^{ik_y y} \sqrt{\frac{1}{L_z}} e^{ik_z z} \tag{S2}$$

where k_y/k_z is the wave vector, L_y/L_z is the size of cubic box. Total probability is 1 in the whole space,

$$\iiint \varphi\varphi^* dxdydz = 1 \tag{S3}$$

where φ^* is the complex conjugate of φ.

Substituting Eq. (S2) into (S3) gives,

$$\begin{aligned} &\int_0^{L_y} \sqrt{\frac{1}{L_y}} e^{ik_y y} \sqrt{\frac{1}{L_y}} e^{-ik_y y} dy = 1 \\ &\int_0^{L_z} \sqrt{\frac{1}{L_z}} e^{ik_z z} \sqrt{\frac{1}{L_z}} e^{-ik_z z} dz = 1 \end{aligned} \tag{S4}$$

Then the electron density depends only on the x-direction wave function, as it is defined in Eq. (9) of the manuscript.

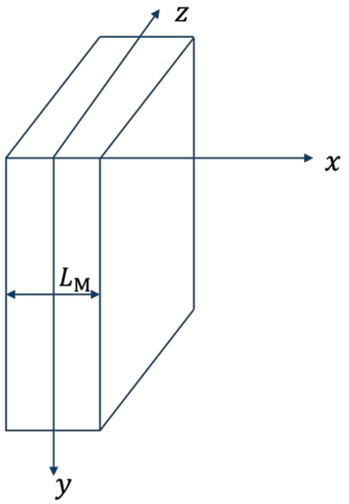

Figure S1 Schematic diagram of Jellium slab model with thickness of L_M

Note 2: derivation of Poisson-Boltzmann equation

The grand potential Ω of the electric double layer is obtained by a Legendre transformation of its Helmholtz free energy F,

$$\Omega = F - \int d^3r \left(n_e \tilde{\mu}_e + \sum_{i=\mathrm{a,c,s}} n_i \tilde{\mu}_i \right) \tag{S5}$$

where $\tilde{\mu}_e$ and $\tilde{\mu}_i$ denote electrochemical potentials of electrons and solution component i, n_e and n_i are their number densities, and the subscripts a, c, and s denote cations, anions and solvent molecules.

The Helmholtz free energy of the electric double layer can be divided into a quantum mechanical part, a classical part and their interactions,

$$F = F_{\mathrm{Q}} + F_{\mathrm{C}} + F_{\otimes} \tag{S6}$$

where F_{Q} describes the quantum-mechanical behavior of the inhomogeneous electron gas, F_{C} describes classical behavior of charged particles and $F_{\otimes}$ describes interactions between the electron gas and classical charged particles. In the following, we introduce each component of F.

According to the Kohn-Sham scheme, we divide F_{Q} as follows,

$$F_{\mathrm{Q}} = T_{\mathrm{ni}}[n_e, \nabla n_e, \ldots] + U_{\mathrm{XC}}[n_e, \nabla n_e, \ldots] \tag{S7}$$

where T_{ni} is the kinetic energy of electrons, U_{XC} is the exchange-correlation energy.

The kinetic energy of electrons can be written as a general form,

$$T_{\mathrm{ni}} = \int d^3r \, t_{ni} \tag{S8}$$

where t_{ni} is the volumetric kinetic energy depending on specific kinetic energy functional, such as the Thomas-Fermi-von Weizsäcker and Pauli-Gaussian kinetic energy functionals, which will be expanded in next section.

The exchange-correlation energy U_{XC} can be written as,

$$U_{\mathrm{XC}} = \int d^3r \, v_{\mathrm{XC}} \tag{S9}$$

where v_{XC} is the volumetric exchange-correlation energy described by a local density approximation, namely, the Dirac-Wigner functional,

$$v_{\mathrm{XC}}(x) = e_{\mathrm{au}} a_0 \left(\frac{4}{3} c_3 n_e^{1/3}(x) + c_4 n_e^{1/3}(x) \frac{\left(\frac{4}{3} c_5 + a_0 n_e^{1/3}(x)\right)}{\left(c_5 + a_0 n_e^{1/3}(x)\right)^2} \right) \tag{S10}$$

where $e_{\mathrm{au}} = \frac{e_0^2}{4\pi\epsilon_0 a_0}$ is the atomic energy with Bohr radius a_0 and vacuum permittivity ϵ_0. The first term in the bracket, with $c_3 = -\frac{3}{4}\left(\frac{3}{\pi}\right)^{1/3}$ and electron density n_{e}, represents the exchange potential of a uniform electron gas; the second term, with $c_4 = -0.056$, $c_5 = 0.079$, is Wigner's expression for the correlation potential [1].

While for the classical part, according to our previous work [2], F_{C} can be described as,

$$F_{\mathrm{C}} = \int d^3 r \, f_{\mathrm{C}} \tag{S11}$$

where f_{C} is the volumetric Helmholtz free energy,

$$f_{\mathrm{C}} = -\frac{1}{2}\epsilon_{\mathrm{op}}(\nabla\phi)^2 + + \sum_{i=1}^{N_C} n_i \left(\delta(i \in \mathrm{M}) q_i \phi - \delta(i \in \mathrm{S}) \beta^{-1} \ln \frac{\sinh(\beta p_i |\nabla\phi|)}{\beta p_i |\nabla\phi|} \right)$$
$$+ \sum_{i=1}^{N_C} \beta^{-1} n_i \left(\ln\left(n_i \Lambda_i^3\right) - 1 \right) + \Phi_{\mathrm{ex}} + (n_{cc} - n_e) e_0 \phi \tag{S12}$$

where the first term is the self-energy of the electric field with ϵ_{op} being the optical permittivity. The second term represents the potential energies of charged particles in solution due to particle-particle coulombic interactions, with $\beta = \frac{1}{k_{\mathrm{B}} T}$ and p_i the dipole moment. The symbol $\delta(i \in \mathrm{M})$ is equal to one for monopolar (M) charged particles, i.e. cations and anions, and zero otherwise, $\delta(i \in S)$ is equal to one for dipolar solvent molecules (S) and zero otherwise. The third term represents the Gibbs free energy of an ideal-gas reference system with Λ_i being the thermal wavelength of particle. The fourth term Φ_{ex} is the excess Gibbs free energy when the reference system deviates from an ideal gas term. The fifth term represents the Hartree energy of electrons and cationic cores of the electrode. Here, Φ_{ex} is described at the level of the Bikerman's lattice-gas theory. Then we obtain the excess chemical potential μ^{ex},

$$\mu_i^{ex} = \frac{\partial \Phi_{\mathrm{ex}}}{\partial n_i} = k_{\mathrm{B}} T \ln \frac{1}{1 - \sum_{i=1}^{N_C} n_i a^3} \tag{S13}$$

where a is the particle size.

The potential energy of the metal-electrolyte interaction is described using the Morse potential,

$$F_{\otimes} = \sum_{i=1}^{N_C} \int d^3r\, n_i w_i \tag{S14}$$

with w_i characterizing short-range interactions between the metal and solution particles, for which we simply use a Morse potential with only its repulsive part to prevent solution particles penetrating into the metal,

$$w_i = D_i \exp\big(-2\beta_i (d - d_i)\big) \tag{S15}$$

with β_i being the coefficient controlling the width of the potential well, and d and d_i denote the distances from the position and the equilibrium position of electrolyte particle to the metal surface, respectively.

Substituting Eq. (S6)-(S14) into Eq. (S5) gives the volumetric grand potential g of the electric double layer,

$$\begin{aligned} g &= t_{\text{in}} + v_{\text{XC}} \\ &+ (n_{cc} - n_e) e_0 \phi - \frac{1}{2} \epsilon_{\text{op}} (\nabla\phi)^2 + \\ &\sum_{i=1}^{N_C} n_i \left(w_i + \delta(i \in \text{M}) q_i \phi - \delta(i \in \text{S}) \beta^{-1} \ln \frac{\sinh\big(\beta p_i |\nabla\phi|\big)}{\beta p_i |\nabla\phi|} \right) \\ &+ \sum_{i=1}^{N_C} \beta^{-1} n_i \big(\ln\big(n_i \Lambda_i^3\big) - 1\big) + \Phi_{\text{ex}} \\ &- n_e \tilde{\mu}_e - \sum_{i=1}^{N_\text{C}} n_i \tilde{\mu}_i \end{aligned} \tag{S16}$$

Variational analysis of g in terms of ϕ gives,

$$\frac{\partial g}{\partial \phi} - \nabla\left(\frac{\partial g}{\partial \nabla\phi}\right) = 0 \tag{S17}$$

leading to

$$-\nabla(\epsilon_{\text{eff}} \nabla\phi) = e_0 (n_{cc} - n_e) + e_0 (q_c n_c - q_a n_a) \tag{S18}$$

which is the Poisson equation with an effective dielectric constant,

$$\epsilon_{\mathrm{eff}} = \epsilon_{\mathrm{op}} + \sum_{i=1}^{N_{\mathrm{C}}} \frac{\delta(i \in \mathrm{S}) n_i p_i}{|\nabla\phi|} \left[\cosh\left(\beta p_i |\nabla\phi|\right) - \frac{1}{\beta p_i |\nabla\phi|}\right] \tag{S19}$$

Next, we need to derive expressions of n_i as functions of ϕ. Variational analysis of g in terms of charged particles in solution gives,

$$w_i + \delta(i \in \mathrm{M}) q_i \phi - \delta(i \in \mathrm{S}) \beta^{-1} \ln \frac{\sinh\left(\beta p_i |\nabla\phi|\right)}{\beta p_i |\nabla\phi|} + \beta^{-1} \ln\left(n_i \Lambda_i^3\right) + \mu_i^{\mathrm{ex}} - \tilde{\mu}_i = 0 \tag{S20}$$

Then we obtain,

$$\frac{n_i \Lambda_i^3}{1 - \sum_{i=1}^{N_{\mathrm{C}}} n_i a^3} = \theta_i \exp(\beta \tilde{\mu}_i) \tag{S21}$$

where θ_i is the thermodynamic factor,

$$\theta_i = \exp\left(-\beta\left(\delta(i \in \mathrm{M}) q_i \phi - \delta(i \in \mathrm{S}) \beta^{-1} \ln \frac{\sinh\left(\beta p_i |\nabla\phi|\right)}{\beta p_i |\nabla\phi|} + w_i\right)\right) \tag{S22}$$

In the solution bulk, $\theta_i = 1$ and $\tilde{\mu}_i$ is uniform in the electrolyte solution. Combining these two conditions gives,

$$\frac{n_i \Lambda_i^3}{1 - \sum_{i=1}^{N_{\mathrm{C}}} n_i a^3} = \theta_i \frac{n_i^{\mathrm{bulk}} \Lambda_i^3}{1 - \sum_{i=1}^{N_{\mathrm{C}}} n_i^{\mathrm{bulk}} a^3} \tag{S23}$$

Then we obtain,

$$n_i = n_i^{\mathrm{bulk}} \frac{\theta_i}{1 + \sum_{i=1}^{N_{\mathrm{C}}} \chi_i (\theta_i - 1)} \tag{S24}$$

where $\chi_{\mathrm{i}} = n_i^{\mathrm{bulk}} a^3$ is the bulk volume fraction.

Eq. (S24) can be extended to scenarios of unequal sizes,

$$n_i = n_i^{\mathrm{bulk}} \frac{\theta_i}{\Omega} \tag{S25}$$

where $\Omega = \sum_{i=1}^{N_{\mathrm{C}}} \chi_i \gamma_i (\theta_i - 1)$ is the normalization factor, and γ_i is the relative size of particles of type *i* referenced to a.

Neglecting the solvent molecules and dipole moment, we obtain the Poisson-Boltzmann equation used in the manuscript,

$$-\nabla\left(\epsilon_{\mathrm{op}}\nabla\phi(x)\right) = e_0(n_{cc} - n_e) + e_0(q_c n_c - q_a n_a) \tag{S26}$$

where

$$n_{\mathrm{c,a}} = n_{\mathrm{c,a}}^{\mathrm{bulk}} \frac{\exp\left(-\frac{\left(w_{\mathrm{c,a}} \pm e_0 q_{\mathrm{c,a}}\phi\right)}{k_{\mathrm{B}}T}\right)}{\chi_{\mathrm{c}}\exp\left(-\frac{(e_0 q_{\mathrm{c}}\phi + w_{\mathrm{c}})}{k_{\mathrm{B}}T}\right) + \chi_{\mathrm{a}}\exp\left(\frac{e_0 q_{\mathrm{a}}\phi - w_{\mathrm{a}}}{k_{\mathrm{B}}T}\right) + 1 - \chi_{\mathrm{c}} - \chi_{\mathrm{a}}} \tag{S27}$$

with $\chi_{\mathrm{c,a}} = n_{\mathrm{c,a}}^{\mathrm{bulk}} a^3$ being the bulk volume fraction of cations/anions.

Note 3: derivation of equation of electron density using Thomas−Fermi−von Weizsäcker kinetic energy functional

The Thomas−Fermi−von Weizsäcker kinetic energy functional is defined as[3, 4],

$$T_{\mathrm{ni}}^{\mathrm{TFvW}} = \int d^3r\, t_{\mathrm{ni}}^{\mathrm{TFvW}} \tag{S28}$$

where $t_{\mathrm{ni}}^{\mathrm{TFvW}}$ is the volumetric Thomas−Fermi−von Weizsäcker kinetic energy,

$$t_{\mathrm{ni}}^{\mathrm{TFvW}} = e_{au} a_0^{-3} t_{\mathrm{TF}}(1 + \theta_T s^2) \tag{S29}$$

where $t_{\mathrm{TF}} = \frac{3}{10}(3\pi^2)^{\frac{2}{3}} n_{\mathrm{e}}^{\frac{5}{3}}$ is Thomas-Fermi (TF) kinetic energy, $s = \frac{|\nabla n_{\mathrm{e}}|}{2(3\pi^2)^{\frac{1}{3}} n_{\mathrm{e}}^{\frac{4}{3}},}$ is the reduced gradient term, θ_{T} is a gradient coefficient tuning the contribution of the gradient term and e_{au} is the atomic energy.

Substituting Eq. (S29) into the grand potential in Eq. (S16) and conducting a variational analysis of g in terms of n_e give,

$$\frac{\partial g}{\partial n_e} - \nabla\left(\frac{\partial g}{\partial \nabla n_e}\right) = 0 \tag{S30}$$

leading to,

$$\nabla\left(\frac{\partial\left(t_{\mathrm{ni}}^{\mathrm{TFvW}} + v_{\mathrm{XC}}\right)}{\partial \nabla n_e}\right) = \frac{\partial\left(t_{\mathrm{ni}}^{\mathrm{TFvW}} + v_{\mathrm{XC}}\right)}{\partial n_e} - e_0\phi - \tilde{\mu}_e \tag{S31}$$

Using the dimensionless variables, $\bar{n}_e = n_e a_0^3$ and $\bar{\nabla} = a_0\nabla$, we obtain,

$$\bar{\nabla}\left(\frac{\partial\left(t_{\mathrm{ni}}^{\mathrm{TFvW}}+v_{\mathrm{XC}}\right)}{\partial\bar{\nabla}\bar{n}_e}\right)=\frac{\partial\left(t_{\mathrm{ni}}^{\mathrm{TFvW}}+v_{\mathrm{XC}}\right)}{\partial\bar{n}_e}-a_0^{-3}(e_0\phi+\tilde{\mu}_e) \tag{S32}$$

With some reorganization, we obtain final controlling equation for the electron density,

$$\bar{\nabla}\bar{\nabla}\bar{n}_{\mathrm{e}}=\frac{20}{3}\bar{n}_{\mathrm{e}}\frac{1}{\theta_{\mathrm{T}}}\left(\frac{\partial t_{\mathrm{TF}}}{\partial\bar{n}_{\mathrm{e}}}+\frac{\partial v_{\mathrm{XC}}}{\partial\bar{n}_{\mathrm{e}}}-\frac{e_0\phi+\tilde{\mu}_{\mathrm{e}}}{e_{\mathrm{au}}}\right)+\frac{2}{3}\frac{1}{\bar{n}_{\mathrm{e}}}\left(\bar{\nabla}\bar{n}_e\right)^2 \tag{S33}$$

Note 4: derivation of equation of electron density using Pauli-Gaussian kinetic energy functional

The Pauli-Gaussian kinetic energy functional is defined as[5],

$$T_{\mathrm{ni}}^{\mathrm{PG}}=T_s^{\mathrm{w}}+\int t_{\mathrm{TF}}\, e^{-\mu_{PG}s^2}d^3r \tag{S34}$$

where $T_s^{\mathrm{w}}=\theta_{\mathrm{T}}s^2\int t_{\mathrm{TF}}d^3r$ is the von Weizsäcker kinetic energy and μ_{PG} is an empirical parameter to be calibrated.

Then we obtain the volumetric Pauli-Gaussian kinetic energy,

$$t_{\mathrm{ni}}^{\mathrm{PG}}=t_{\mathrm{TF}}\theta_{\mathrm{T}}s^2+\int t_{\mathrm{TF}}\, e^{-\mu_{PG}s^2} \tag{S35}$$

Substituting Eq. (S35) into the grand potential in Eq. (S16) and conducting a variational analysis of g in terms of n_e give,

$$\frac{\partial g}{\partial n_e}-\nabla\left(\frac{\partial g}{\partial\nabla n_e}\right)=0 \tag{S36}$$

leading to,

$$\nabla\left(\frac{\partial\left(t_{\mathrm{ni}}^{\mathrm{PG}}+v_{\mathrm{XC}}\right)}{\partial\nabla n_e}\right)=\frac{\partial\left(t_{\mathrm{ni}}^{\mathrm{PG}}+v_{\mathrm{XC}}\right)}{\partial n_e}-e_0\phi-\tilde{\mu}_e \tag{S37}$$

Using the dimensionless variables, $\bar{n}_e=n_ea_0^3$ and $\bar{\nabla}=a_0\nabla$, we obtain,

$$\bar{\nabla}\left(\frac{\partial\left(t_{\mathrm{ni}}^{\mathrm{PG}}+v_{\mathrm{XC}}\right)}{\partial\bar{\nabla}\bar{n}_e}\right)=\frac{\partial\left(t_{\mathrm{ni}}^{\mathrm{PG}}+v_{\mathrm{XC}}\right)}{\partial\bar{n}_e}-a_0^{-3}(e_0\phi+\tilde{\mu}_e) \tag{S38}$$

With some reorganization, we obtain final controlling equation for the electron density,

$$\bar{\nabla}\bar{\nabla}\bar{n}_{\mathrm{e}}=\frac{20}{3}\bar{n}_{\mathrm{e}}\frac{1}{\left(\theta_{\mathrm{T}}-\mu_{\mathrm{PG}}e^{-\mu_{\mathrm{PG}}s^2}\right)}\left(e^{-\mu_{\mathrm{PG}}s^2}\frac{\partial t_{\mathrm{TF}}}{\partial\bar{n}_{\mathrm{e}}}+\frac{\partial v_{\mathrm{XC}}}{\partial\bar{n}_{\mathrm{e}}}-\frac{(e_0\phi+\tilde{\mu}_e)}{e_{\mathrm{au}}}\right)+ \tag{S39}$$

$$\frac{\left(\theta_{\mathrm{T}}+\frac{2}{3}\mu_{\mathrm{PG}}e^{-\mu_{\mathrm{PG}}s^2}+\frac{16}{3}\mu_{\mathrm{PG}}{}^2 s^2 e^{-\mu_{\mathrm{PG}}s^2}\right)}{2\overline{n}_{\mathrm{e}}(\theta_{\mathrm{T}}-\mu_{\mathrm{PG}}e^{-\mu_{\mathrm{PG}}s^2})}\left(\overline{\nabla}\overline{n}_{\mathrm{e}}\right)^2$$

Note 5: derivation of the definition of electron density

Here we provide a detailed derivation for the definition of electron density in Eq. (7) of the manuscript.

From the formulation of 1D Jellium model in the last section, we can rewrite the wave function with plane waves explicitly included in the y and z directions and a real-space function $\varphi_i(x)$ describing the variation normal to the surface,

$$\varphi=\frac{e^{-ik_y y}}{\sqrt{L_y}}\frac{e^{-ik_z z}}{\sqrt{L_z}}\varphi_i(x) \tag{S40}$$

The total energy is,

$$\begin{aligned}\varepsilon_{\mathrm{tot}}&=\varepsilon_i+\frac{\hbar^2}{2m}K^2\\ K^2&=k_y^2+k_z^2\end{aligned} \tag{S41}$$

where the second term is the total energy stemming from y and z directions.

The electron density is defined as,

$$n_{\mathrm{e}}(x)=2\sum_{k_y,k_z}\frac{1}{S}|\varphi_i(x)|^2 f_0(\varepsilon_{\mathrm{tot}}-\varepsilon_{\mathrm{F}}) \tag{S42}$$

where 2 accounts for the electron spin, *S* is the area of the Fermi surface, and f_0 is the Fermi-Dirac distribution defined as,

$$f_0(\varepsilon_{tot}-\varepsilon_F)=\frac{1}{1+\exp\left(\frac{\varepsilon_{\mathrm{tot}}-\varepsilon_{\mathrm{F}}}{k_B T}\right)} \tag{S43}$$

Using the standard-wave counting,

$$\frac{1}{S}\sum_{k_y,k_z}\rightarrow=\frac{1}{(2\pi)^2}\int d^2K \tag{S44}$$

Since the integration depends only on K^2, we switch to polar coordinates,

$$d^2K=KdKd\theta,\qquad \theta\in[0,2\pi] \tag{S45}$$

Performing the angular integral gives,

$$\int d^2K=2\pi\int_0^\infty KdK \tag{S46}$$

Substituting Eqs. (S41), (S43), (S44) and (S46) into Eq. (S42) gives,

$$n_e(x) = \frac{1}{\pi}\sum_i \overline{\varphi}_i^2 \int_0^{\infty} KdK\, f_0\left(\varepsilon_i + \frac{\hbar^2}{2m}K^2 - \varepsilon_F\right) \tag{S47}$$

Defining $u = \frac{\hbar^2}{2mk_BT}K^2$, we obtain $KdK = \frac{mk_BT}{\hbar^2}du$. Then Eq. (S47) can be rewritten as,

$$n_e(x) = \frac{mk_{\mathrm{B}}T}{\hbar^2\pi}\sum_i \overline{\varphi}_i^2 \int_0^{\infty} \frac{du}{1 + \exp\left(u + \frac{\varepsilon_i - \varepsilon_F}{k_BT}\right)} \tag{S48}$$

Using the elementary identity,

$$\int_0^{\infty} \frac{du}{1 + \exp(u + \zeta)} = \ln(1 + e^{-\zeta}) \tag{S49}$$

we obtain,

$$n_e(x) = \frac{mk_{\mathrm{B}}T}{\hbar^2\pi}\sum_i \overline{\varphi}_i^2 \ln(1 + e^{(\varepsilon_{\mathrm{F}} - \varepsilon_i)/k_{\mathrm{B}}T}) \tag{S50}$$

Using the dimensionless variables defined in the manuscript, we obtain the dimensionless form of the electron density[6, 7],

$$\overline{n}_{\mathrm{e}} = a_0^2 \frac{mk_{\mathrm{B}}T}{\hbar^2\pi}\sum_i \overline{\varphi}_i^2 \ln(1 + e^{(\varepsilon_{\mathrm{F}} - \varepsilon_i)/k_{\mathrm{B}}T}) \tag{S51}$$

Note 6: theoretical formulation of boundary conditions for Kohn-Sham-Poisson-Boltzmann model

Herein we give a detailed theoretical formulation of boundary conditions for the Kohn-Sham-Poisson-Boltzmann model.

1. Boundary condition for the Kohn-Sham equation

In the bulk electrolyte region, the effective Kohn–Sham potential becomes spatially uniform, $v_{\mathrm{eff}}(x) \to v_{\mathrm{sol}}$, and the eigenvalues of the occupied electronic states satisfy $\varepsilon_{\mathrm{i}} < v_{\mathrm{sol}}$. In this asymptotic region, the one-dimensional Kohn-Sham equation is reduced to,

$$\left[-\frac{\hbar^2}{2m_{\mathrm{e}}}\frac{d^2}{dx^2} + v_{\mathrm{sol}}\right]\varphi_i(x) = \varepsilon_{\mathrm{i}}\varphi_i(x) \tag{S52}$$

Reorganizing Eq. (S52) as an ordinary differential equation gives,

$$\frac{d^2\varphi_i}{dx^2} = \kappa_i^2\varphi_i \tag{S53}$$

with $\kappa_i = \sqrt{2\frac{m_e}{\hbar^2}(v_{\mathrm{sol}} - \varepsilon_{\mathrm{i}})}$>0.

The general solution of Eq. (S53) can be expressed as,

$$\varphi_i = A_i e^{-\kappa_i x} + B_i e^{k_i x} \tag{S54}$$

where A_i and B_i are coefficients determined by the requirement of the normalization and boundary conditions.

For a symmetrical system in $|x|$, we can rewrite Eq. (S54) as,

$$\varphi_i = A_i e^{-\kappa_i |x|} + B_i e^{k_i |x|} \tag{S55}$$

To ensure normalizability of wave functions, then we must remove the growing branch of Eq. (S55), namely, $B_i = 0$.

Therefore, the solution of φ_i is expressed as,

$$\varphi_i = A_i e^{-\kappa_i |x|} \tag{S56}$$

In solution bulk, $|x| \to +\infty$, we obtain the asymptotic value of φ_i,

$$\varphi_i = 0 \tag{S57}$$

which is the boundary condition used for the Kohn-Sham equation.

2. Boundary condition for the Poisson-Boltzmann equation

The one-dimensional Poisson-Boltzmann equation is expressed as,

$$-\nabla\big(\epsilon_{\mathrm{op}}(x)\nabla\phi(x)\big) = \rho_{\mathrm{tot}} \tag{S58}$$

with $\rho_{\mathrm{tot}} = e_0(n_{\mathrm{cc}} - n_{\mathrm{e}}) + e_0(q_{\mathrm{c}}n_{\mathrm{c}} - q_{\mathrm{a}}n_{\mathrm{a}})$ being the total net charge from electron, cationic core, cation and anion.

In the bulk solution, $|x| \to +\infty$, $\epsilon_{\mathrm{op}}(x) = \epsilon_{\mathrm{sol}}$ and $\rho_{\mathrm{tot}} = 0$. Then Eq. (S58) is reduced to,

$$-\nabla\big(\epsilon_{\mathrm{sol}}\nabla\phi(x)\big) = 0 \tag{S59}$$

Because ϵ_{sol} is a constant in the bulk solution, we can rewrite Eq. (S59) as,

$$\frac{d^2\phi(x)}{dx^2} = 0 \tag{S60}$$

The general solution of $\phi(x)$ can be expressed is,

$$\phi(x) = ax + b \tag{S61}$$

with a and b are two coefficients.

The electric field is zero in the bulk solution, which means,

$$-\frac{d\phi(x)}{dx} = 0, a = 0 \tag{S62}$$

Then the solution of $\phi(x)$ is reduced to,

$$\phi(x) = b \tag{S63}$$

The electrostatic potential is defined relative to a reference value. By choosing the electrostatic potential in the bulk solution as the zero reference, we obtain,

$$\phi(x) = 0 \tag{S64}$$

which is the boundary condition used for the Poisson-Boltzmann equation.

Note 7: discretization of Poisson-Boltzmann equation with finite element method

Multiplying Eq. (15) in the manuscript by a test function v and using integration by parts, we obtain the weak form of the Poisson-Boltzmann equation,

$$\int \bar{\epsilon}_{\mathrm{op}} \bar{\nabla} \bar{\phi}_i \cdot \bar{\nabla} v d\bar{x} = \int f\, v d\bar{x} \tag{S65}$$

where $f = \frac{4\pi e_{\mathrm{au}}}{k_{\mathrm{B}}T}\left(\bar{n}_{\mathrm{cc}} - \bar{n}_{\mathrm{e}}\right) + \frac{4\pi e_{\mathrm{au}}}{k_{\mathrm{B}}T}\left(q_{\mathrm{c}}\bar{n}_{\mathrm{c}} - q_{\mathrm{a}}\bar{n}_{\mathrm{a}}\right)$ is the source term.

Approximating $\bar{\phi}_i$ with the linear Lagrangian basis function [8] $\bar{\phi}_i = \sum_{i=1}^{n} c_i \bar{\phi}_i$ gives,

$$\sum_{i=1}^{n} c_i \int \bar{\epsilon}_{\mathrm{op}} \bar{\nabla} \bar{\phi}_i \cdot \bar{\nabla} v d\bar{x} = \int f\, v d\bar{x} \tag{S66}$$

where c_i are coefficients to be determined.

Furthermore, using the Galerkin method [9] to describe the test function v, namely, $v = \bar{\phi}_j$, we obtain,

$$\sum_{i=1}^{n} c_i \int \bar{\epsilon}_{\text{op}} \bar{\nabla}\bar{\phi}_i \cdot \bar{\nabla}\bar{\phi}_j d\bar{x} = \int f\, \bar{\phi}_j d\bar{x} \tag{S67}$$

which can be rewritten as,

$$\boldsymbol{K_{ij} c_i} = \boldsymbol{F_i} \tag{S68}$$

where $\boldsymbol{K_{ij}} = \int \bar{\epsilon}_{\text{op}} \bar{\nabla}\bar{\phi}_i \cdot \bar{\nabla}\bar{\phi}_k d\bar{x}$ is the so-called stiffness matrix and $\boldsymbol{F_i} = \int f\bar{\phi}_i d\bar{x}$.

$\bar{\epsilon}_{\text{op}}$ varies in the space between the metal and the electrolyte solution. Nonuniform distribution of $\bar{\epsilon}_{\text{op}}$ renders $\boldsymbol{K_{ij}}$ become asymmetrical and difficult to solve. We use an average value $\bar{\epsilon}_{\text{op}}^{i\pm1/2}$ to represent local $\bar{\epsilon}_{\text{op}}$ between $\bar{x}_i$ and $\bar{x}_{i\pm1}$, defined as,

$$\bar{\epsilon}_{\text{op}}^{i\pm1/2} = \frac{\bar{\epsilon}_{\text{op}}(\bar{x}_i) + \bar{\epsilon}_{\text{op}}(\bar{x}_{i\pm1})}{2} \tag{S69}$$

Using a 1D uniform mesh grid, we obtain the local stiffness matrix [8],

$$\boldsymbol{K_{ij}^{\text{local}}} = \bar{\epsilon}_{\text{op}}^{i\pm1/2} \int \frac{1}{d\bar{x}^2} \begin{bmatrix} 1 & -1 \\ -1 & 1 \end{bmatrix} d\bar{x} = \frac{\bar{\epsilon}_{\text{op}}^{i\pm1/2}}{d\bar{x}} \begin{bmatrix} 1 & -1 \\ -1 & 1 \end{bmatrix} \tag{S70}$$

Assembling $\boldsymbol{K_{ij}^{\text{local}}}$ into $\boldsymbol{K_{ij}}$ gives the discretization form of the Poisson-Boltzmann equation,

$$\boldsymbol{K_{ij}} = \begin{cases} \dfrac{\bar{\epsilon}_{\text{op}}^{i-1/2} + \bar{\epsilon}_{\text{op}}^{i+1/2}}{d\bar{x}}, & \text{if } i = j \\ -\dfrac{\bar{\epsilon}_{\text{op}}^{i\pm1/2}}{d\bar{x}} & \text{if } i = |j \pm 1| \\ 0, & \text{otherwise} \end{cases} \tag{S71}$$

$$\boldsymbol{F_i} = f d\bar{x} \tag{S72}$$

$$\boldsymbol{c_i} = \boldsymbol{K_{ij}} \setminus \boldsymbol{F_i} \tag{S73}$$

where the backslash " \ " represents a matrix left-division operation of solving a linear system such as $\boldsymbol{K_{ij} c_i} = \boldsymbol{F_i}$ and $\boldsymbol{c_i}$ is the solution collecting the components of $\bar{\phi}_i$.

Note 8: numerical implementation of boundary conditions

For both the Poisson Boltzmann equation (solved with the finite element method) and the Kohn-Sham equation (solved with the finite difference method), homogeneous Dirichlet boundary conditions (electrostatic potential $\phi = 0$ and wave function $\varphi = 0$) were applied. The specific numerical implementations are as follows:

1. Finite element method for the Poisson-Boltzmann equation

The initial global system after discretization is: $\boldsymbol{Kc} = \boldsymbol{F}$ with $\boldsymbol{K}$ being the stiffness matrix and ***F*** being the source vector.

To enforce ϕ =0 at boundary nodes (index 1 and *N*), the standard essential boundary condition procedure is applied. This modifies the global system as follows:

1) Set the 1st and *N*th row of $\boldsymbol{K}$ to zero and the diagonal element of $\boldsymbol{K}$ to 1, which means $\boldsymbol{K}(1,:) = 0$, $\boldsymbol{K}(1,1) = 1$, $\boldsymbol{K}(N,:) = 0$, and $\boldsymbol{K}(N,N) = 1$.
2) Set the 1st and *N*th elements of the source vector $\boldsymbol{F}$ to 0, which means $\boldsymbol{F}(1) = \boldsymbol{F}(N) = 0$.

2. Finite difference method for the Kohn-Sham equation

The initial global system after discretization is: $\boldsymbol{H\varphi} = \bar{\boldsymbol{\varepsilon}}\boldsymbol{\varphi}$ with ***H*** being the Hamiltonian matrix.

To enforce $\varphi = 0$ at boundary nodes (index 1 and *N*), the corresponding rows and columns are zeroed out, and the diagonal of $\boldsymbol{H}$ is set to 1. Then the Hamiltonian matrix $\boldsymbol{H}$ with incorporated boundary conditions is as follows: $\boldsymbol{H}(1,:) = 0$, $\boldsymbol{H}(1,1) = 1$, $\boldsymbol{H}(N,:) = 0$, and $\boldsymbol{H}(N,N) = 1$.

Note 8: initial guesses of $\bar{\boldsymbol{\phi}}_0$ and $\bar{\boldsymbol{n}}_{\mathbf{e}}^{\mathbf{0}}$

To avoid numerical instability of self-consistent calculation, herein we construct reasonable initial guesses of $\bar{\phi}_0$ and $\bar{n}_{\mathrm{e}}^0$. Specifically, we use error function to achieve a smooth transition of electrostatic potential from metal to solution,

$$\bar{\phi}_0 = \frac{\bar{\phi}_0^{\mathrm{M}}}{2}\,\mathrm{erfc}(|\bar{x}| - L_{\mathrm{M}}) \tag{S74}$$

where erfc is the complementary error function and $\bar{\phi}_0^{\mathrm{M}}$ is the dimensionless electrostatic potential in the metal bulk.

Similarly, $\bar{n}_{\mathrm{e}}^0$ also adopts error function to construct reasonable initial distributions,

$$\bar{n}_{\mathrm{e}}^0 = \frac{\bar{n}_{\mathrm{cc}}}{2}\,\mathrm{erfc}(|\bar{x}| - L_{\mathrm{M}}) \tag{S75}$$

where $\bar{n}_{\mathrm{cc}}$ is the dimensionless cationic core density.

Note 9: estimation of dimensionless cationic core density $\bar{n}_{\mathrm{cc}}$

The cationic core density $\bar{n}_{\mathrm{cc}}$ can be determined from the effective Wigner-Seitz radius or the cubic cell structure. We introduce the two methods in turn and then compare the results between them. The dimensionless effective Wigner-Seitz radius is defined as[10],

$$\bar{r}_s = r_s/a_0 = \left(\frac{3}{4\pi\bar{n}_{\mathrm{cc}}}\right)^{1/3} \quad \text{(S76)}$$

With a simple mathematical transformation, we obtain,

$$\bar{n}_{\mathrm{cc}} = \frac{3}{4\pi\bar{r}_s^3} \quad \text{(S77)}$$

Table S1 gives the values of $\bar{r}_s$ and $\bar{n}_{cc}$ calculated using Eq. (S77) for the first group metals, we notice the minimum value of $\bar{n}_{\mathrm{cc}}$ is about 0.001.

Table S1 Values of $\bar{r}_s$ and $\bar{n}_{\mathrm{cc}}$ for the first group metals

Metal	$\bar{r}_s$	$\bar{n}_{\mathrm{cc}}$
Li	3.25	0.007
Na	3.93	0.0039
K	4.86	0.0021
Rb	5.20	0.0017
Cs	5.62	0.0013

$\bar{n}_{\mathrm{cc}}$, determined from the cubic cell structure, is defined as[2],

$$\bar{n}_{\mathrm{cc}} = N_{\mathrm{atom}} N_{\mathrm{M}} \left(\frac{a_0}{a_{\mathrm{M}}}\right)^3 \quad \text{(S78)}$$

where N_{atom} is the atom number in a cubic cell, N_{M} is the valence electron number in an atom and a_{M} is the lattice constant of the cubic closed-packed cell. For the first group metals, $N_{\mathrm{atom}} = 2$ and $N_{\mathrm{M}} = 1$.

Table S2 presents the values of a_{M} and $\bar{n}_{\mathrm{cc}}$ calculated using Eq. (S78) for the first-group metals. The values are consistent with those derived from the Wigner-Seitz radius, except for Na. This consistency demonstrates that setting 0.001 as the minimum value of $\bar{n}_{\mathrm{cc}}$ is reasonable.

Table S2 Values of a_{M} and $\bar{n}_{\mathrm{cc}}$ for the first group metals

Metal	$a_{\mathrm{M}}/\mathrm{Å}$	$\bar{n}_{\mathrm{cc}}$
Li	3.49	0.007

Na	4.29	0.0037
K	5.23	0.0021
Rb	5.59	0.0017
Cs	6.05	0.0013

Note 10: determination of PZC for the KSPB model at $\bar{n}_{\mathrm{cc}}$=0.01

The PZC is defined as the electrode potential at which the surface free charge is zero[11]. Figure S2 shows the plots of the dimensionless surface free charge $\bar{\sigma}_{\mathrm{free}}$ versus negative electrochemical potential of electrons $-\bar{\mu}_e$. We observe that $\bar{\sigma}_{\mathrm{free}}$ is approximately zero when $-\bar{\mu}_e$ lies in the regime of 3.56 eV to 3.57 eV.

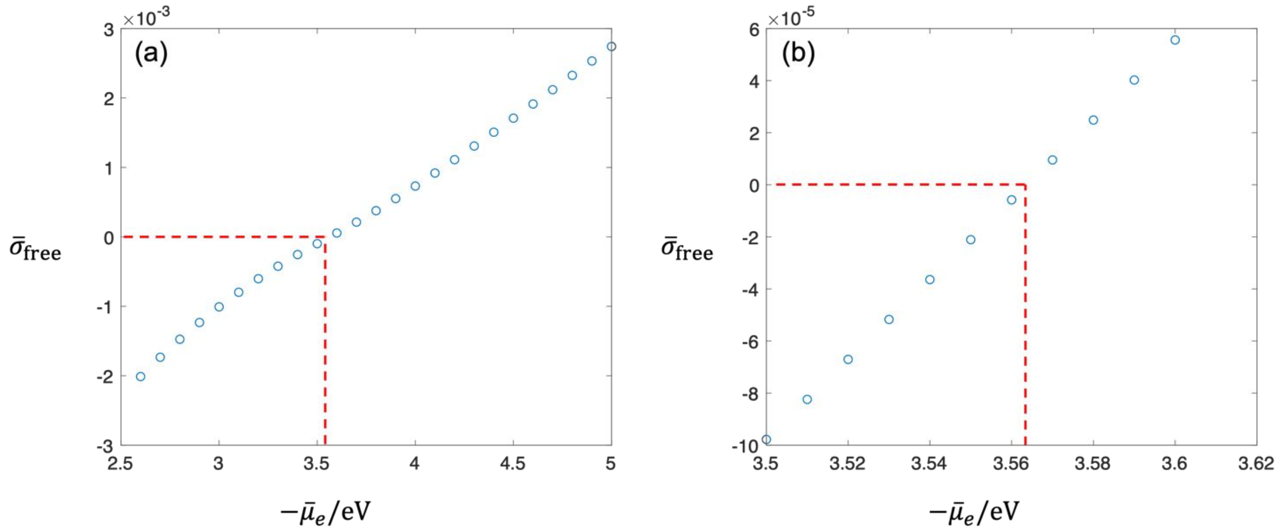

Figure S2 (a) The dimensionless surface free charge versus negative electrochemical potential of electrons, (b) enlarged view of (a) between $-\bar{\mu}_e = 3.5\ \mathrm{eV}$ and $-\bar{\mu}_e = 3.6\ \mathrm{eV}$. Parameters used in calculation are the same as those in Figure 3 of the manuscript.

Note 11: error analysis of TFvW and Pauli-Gaussian kinetic functionals at $\bar{n}_{\mathrm{cc}} = 0.01$

Figure S3 (a) and (b) show the comparison between the DPFT model with TFvW functional and Pauli-Gaussian functional and the Kohn-Sham-Poisson-Boltzmann model in terms of $\tilde{\mu}_e^{\mathrm{PZC}}$. For the DPFT model described using the TFvW functional, $\tilde{\mu}_e^{\mathrm{PZC}}$ becomes more negative with increasing θ_{T}. The TFvW kinetic functional with $\theta_{\mathrm{T}} = 0.7$ gives a better approximation in terms of $\tilde{\mu}_e^{\mathrm{PZC}}$. For the DPFT model described using the Pauli-Gaussian functional, $\tilde{\mu}_e^{\mathrm{PZC}}$ becomes more positive with

increasing μ_{PG}. The Pauli-Gaussian kinetic functional with $\mu_{\mathrm{PG}} = 0.25$ gives a better approximation in terms of $\tilde{\mu}_e^{\mathrm{PZC}}$. To quantify the error of the DPFT model relative to the Kohn-Sham-Poisson-Boltzmann model, we define a mean error as, $\mathrm{Error} = \frac{1}{N_{\mathrm{tot}}}\sum \frac{|C_{\mathrm{dl}}^{\mathrm{DPFT}} - C_{\mathrm{dl}}^{\mathrm{Kohn-Sham-Poisson-Boltzmann}}|}{C_{\mathrm{dl}}^{\mathrm{Kohn-Sham-Poisson-Boltzmann}}}$ with N_{tot} being total number of $C_{\mathrm{dl}}^{\mathrm{DPFT}}$ data points. Figure S3 (c) and (d) give the error in C_{dl} calculated from the DPFT model described using the TFvW functional and Pauli-Gaussion functional relative to the Kohn-Sham-Poisson-Boltzmann model results, respectively. For the TFvW kinetic functional, the error increases with increasing θ_{T}. Therefore, the minimum error occurs at $\theta_{\mathrm{T}} = 0.5$, differing from that in terms of $\tilde{\mu}_e^{\mathrm{PZC}}$. For the Pauli-Gaussian kinetic functional, the error decreases with increasing μ_{PG}. The error is smallest at $\mu_{\mathrm{PG}} = 0.35$, again differing from that in terms of $\tilde{\mu}_e^{\mathrm{PZC}}$.

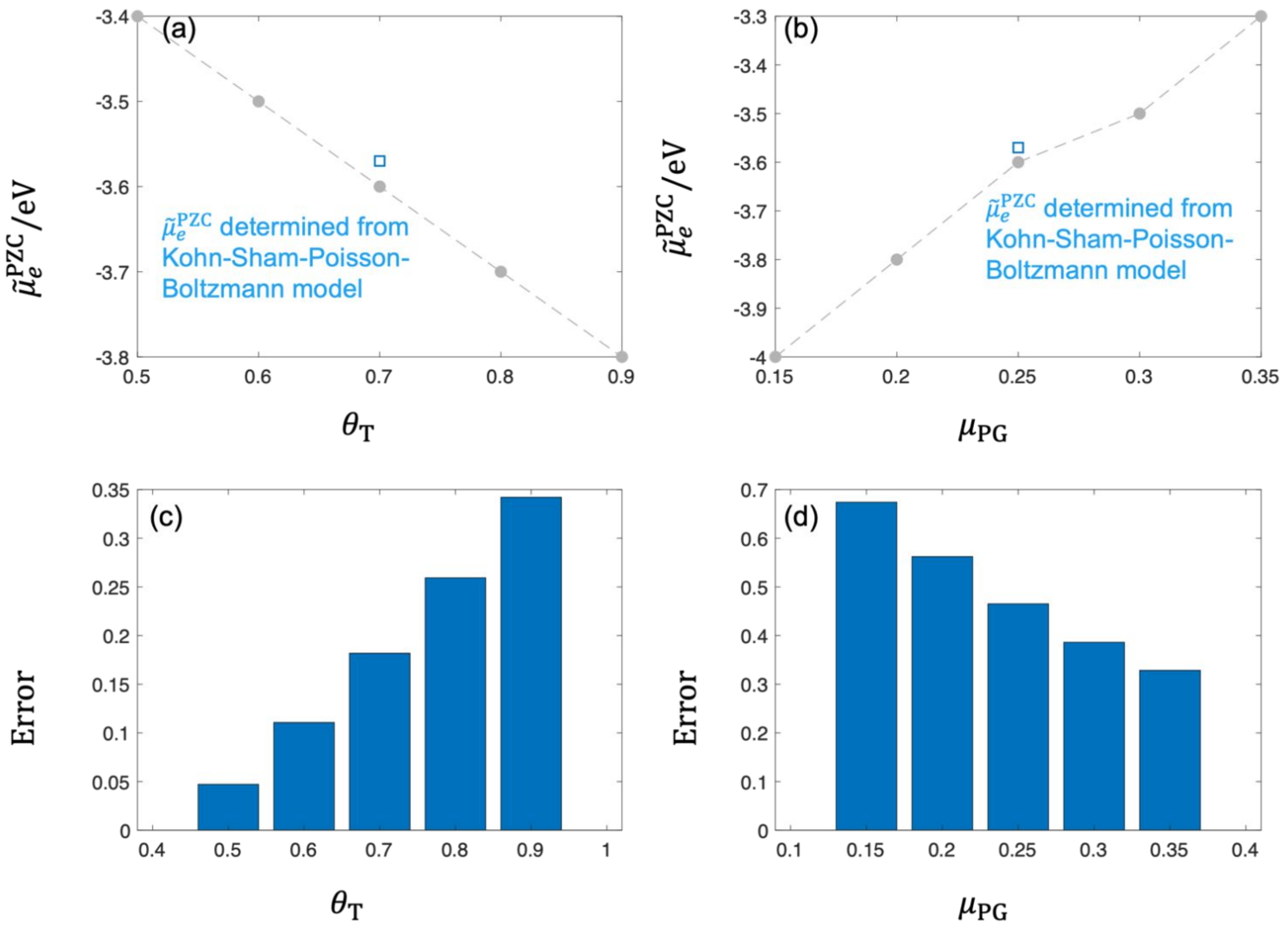

Figure S3 Comparison between DPFT model with (a) TFvW functional, (b) Pauli-Gaussian functional and Kohn-Sham-Poisson-Boltzmann model results in terms of $\tilde{\mu}_e^{\mathrm{PZC}}$, mean error in double-layer capacitance calculated from the DPFT model with (c) TFvW functional, (d) Pauli-Gaussian functional relative to results of the Kohn-Sham-Poisson-Boltzmann model. $\bar{n}_{\mathrm{cc}} = 0.01$, other parameters are the same as those in Figure 3 in the manuscript.

Note 12: error analysis of TFvW and Pauli-Gaussian functionals at $\bar{n}_{\mathrm{cc}} = 0.005$

Figure S4 (a) and (b) show the comparison between the DPFT model with TFvW functional and Pauli-Gaussian functional and the Kohn-Sham-Poisson-Boltzmann model in terms of $\tilde{\mu}_e^{\mathrm{PZC}}$ at $\bar{n}_{\mathrm{cc}} = 0.005$. The TFvW kinetic functional at both $\theta_{\mathrm{T}} = 0.7$ and $\theta_{\mathrm{T}} = 0.8$ gives a better approximation in terms of $\tilde{\mu}_e^{\mathrm{PZC}}$. The Pauli-Gaussian kinetic functional at $\mu_{\mathrm{PG}} = 0.2$ gives a better approximation in terms of $\tilde{\mu}_e^{\mathrm{PZC}}$. Figure S4 (c) and (d) give the mean error in double-layer capacitance calculated from the DPFT model described by the TFvW functional and Pauli-Gaussion functional relative to the Kohn-Sham-Poisson-Boltzmann model results, respectively. For the TFvW kinetic functional, the error is smallest at $\theta_{\mathrm{T}} = 0.5$, differing from that in terms of $\tilde{\mu}_e^{\mathrm{PZC}}$. For the Pauli-Gaussian kinetic functional, the error is smallest at $\mu_{\mathrm{PG}} = 0.35$, again differing from that in terms of $\tilde{\mu}_e^{\mathrm{PZC}}$.

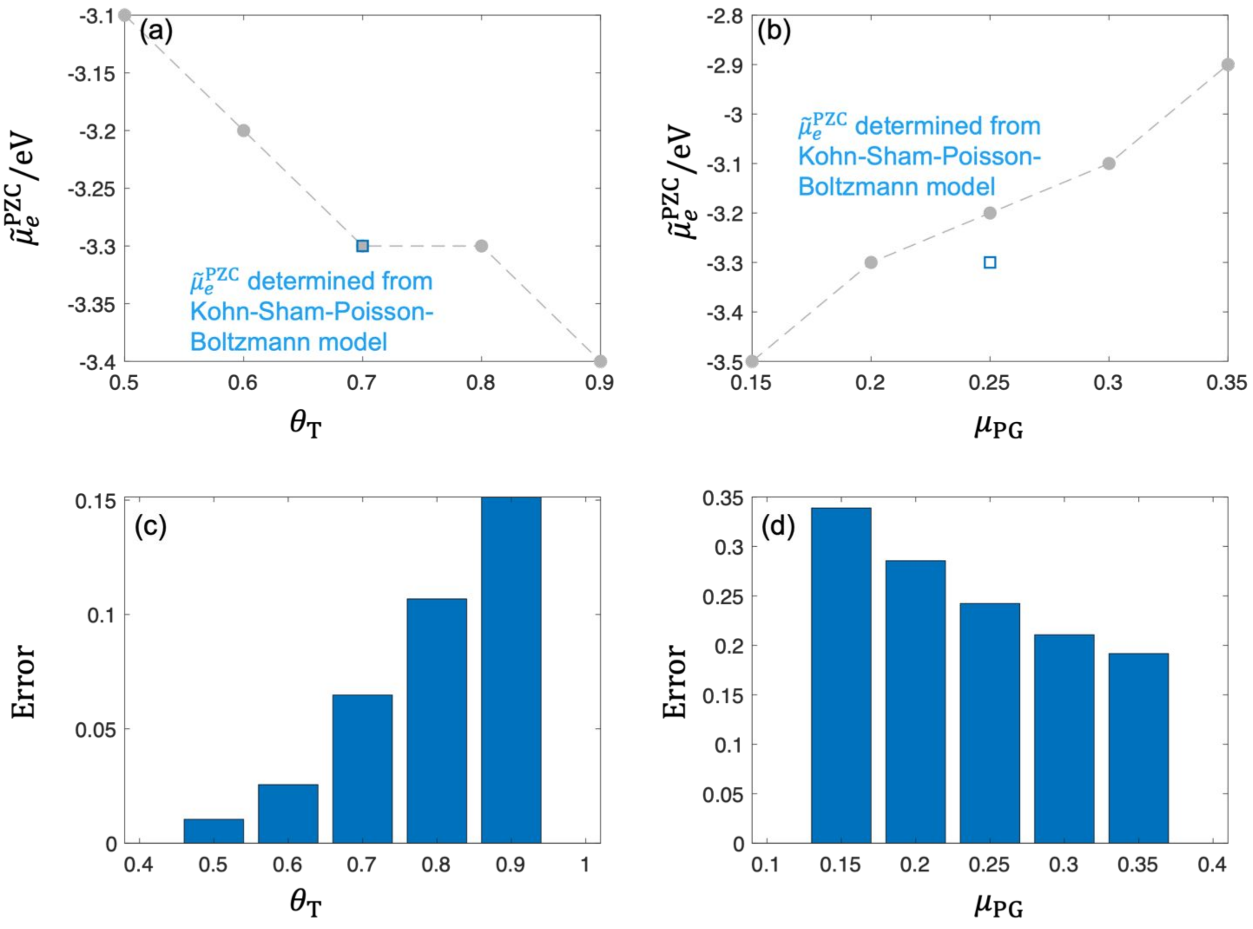

Figure S4 Comparison between DPFT model with (a)TFvW functional, (b) Pauli-Gaussian functional and Kohn-Sham-Poisson-Boltzmann model results in terms of $\tilde{\mu}_e^{\mathrm{PZC}}$, mean error in double layer capacitance calculated from the DPFT model with (c) TFvW functional, (d) Pauli-

Gaussian functional relative to Kohn-Sham-Poisson-Boltzmann model results. $\bar{n}_{\mathrm{cc}} = 0.005$, other parameters are the same as those in Figure 3 in the manuscript.

Reference

(1) Wigner, E. On the interaction of electrons in metals. *Physical Review* **1934**, *46* (11), 1002.
(2) Huang, J. Density-potential functional theory of electrochemical double layers: Calibration on the Ag (111)-KPF6 system and parametric analysis. *Journal of chemical theory and computation* **2023**, *19* (3), 1003–1013.
(3) Thomas, L. H. The calculation of atomic fields. In *Mathematical proceedings of the Cambridge philosophical society*, 1927; Cambridge University Press: Vol. 23, pp 542–548.
(4) Fermi, E. Eine statistische Methode zur Bestimmung einiger Eigenschaften des Atoms und ihre Anwendung auf die Theorie des periodischen Systems der Elemente. *Zeitschrift für Physik* **1928**, *48* (1), 73–79.
(5) Constantin, L. A.; Fabiano, E.; Della Sala, F. Semilocal Pauli–Gaussian kinetic functionals for orbital-free density functional theory calculations of solids. *The journal of physical chemistry letters* **2018**, *9* (15), 4385–4390.
(6) Datta, S. Nanoscale device modeling: the Green's function method. *Superlattices and microstructures* **2000**, *28* (4), 253–278.
(7) Datta, S. *Quantum transport: atom to transistor*; Cambridge university press, 2005.
(8) Zienkiewicz, O. C.; Taylor, R. L.; Zhu, J. Z. *The finite element method: its basis and fundamentals*; Elsevier, 2005.
(9) Fletcher, C. A.; Fletcher, C. *Computational galerkin methods*; Springer, 1984.
(10) Lang, N.; Kohn, W. Theory of metal surfaces: charge density and surface energy. *Physical Review B* **1970**, *1* (12), 4555.
(11) Zhang, L.-L.; Li, C.-K.; Huang, J. A Beginners' Guide to Modelling of Electric Double Layer under Equilibrium, Nonequilibrium and AC Conditions. *Journal of Electrochemistry* **2022**, *28* (2), 2108471.
(12) Perdew, J. P.; Burke, K.; Ernzerhof, M. Generalized gradient approximation made simple. *Physical review letters* **1996**, *77* (18), 3865.
